# Noncanonical genomic imprinting in the monoamine system determines naturalistic foraging and brain-adrenal axis functions

**DOI:** 10.1016/j.celrep.2022.110500

**Published:** 2022-03-08

**Authors:** Paul J. Bonthuis, Susan Steinwand, Cornelia N. Stacher Hörndli, Jared Emery, Wei-Chao Huang, Stephanie Kravitz, Elliott Ferris, Christopher Gregg

**Affiliations:** 1 Department of Comparative Biosciences, University of Illinois at Urbana-Champaign College of Veterinary Medicine, Urbana, IL, USA; 2 Department of Neurobiology, University of Utah School of Medicine, Room 408B, Biopolymers Research Building, Bld. 570, 20 South 2030 East, Salt Lake City, UT 84112, USA; 3 Department of Human Genetics, University of Utah School of Medicine, Room 408B, Biopolymers Research Building, Bld. 570, 20 South 2030 East, Salt Lake City, UT 84112, USA; 4 These authors contributed equally; 5 Lead contact

## Abstract

Noncanonical genomic imprinting can cause biased expression of one parental allele in a tissue; however, the functional relevance of such biases is unclear. To investigate ethological roles for noncanonical imprinting in *dopa decarboxylase* (*Ddc*) and *tyrosine hydroxylase* (*Th*), we use machine learning to decompose naturalistic foraging in maternal and paternal allele mutant heterozygous mice. We uncover distinct roles for the maternal versus paternal alleles on foraging, where maternal alleles affect sons while daughters are under paternal allelic control. Each parental allele controls specific action sequences reflecting decisions in naive or familiar contexts. The maternal *Ddc* allele is preferentially expressed in subsets of hypothalamic GABAergic neurons, while the paternal allele predominates in subsets of adrenal cells. Each *Ddc* allele affects distinct molecular and endocrine components of the brain-adrenal axis. Thus, monoaminergic noncanonical imprinting has ethological roles in foraging and endocrine functions and operates by affecting discrete subsets of cells.

## In brief

Bonthuis et al. uncover parental controls over foraging decisions and actions, as well as brain-adrenal axis physiology, through allele-specific expression of tyrosine hydroxylase and/or dopa decarboxylase enzymes in specific brain and adrenal cells of offspring. Maternal alleles determine phenotypes in sons, while paternal alleles affect daughters.

## Graphical Abstract

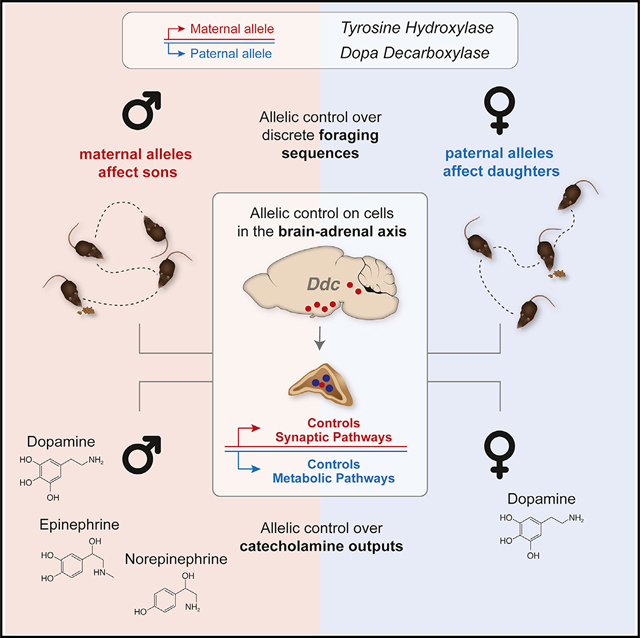

## INTRODUCTION

Genomic imprinting is a heritable form of allele-specific epigenetic gene regulation that causes preferential expression of the maternally or paternally derived allele for some genes in mammals (Bartolomei and Ferguson-Smith, 2011). Multiple theories for the evolution of imprinting have been proposed (Haig, 2004; Spencer and Clark, 2014). However, the functions of imprinting and relative roles of maternally versus paternally expressed imprinted genes (MEGs and PEGs, respectively) are not well understood. We (Bonthuis et al., 2015) and others (Andergassen et al., 2017; Babak et al., 2015; Crowley et al., 2015; Perez et al., 2015) previously uncovered “noncanonical” imprinting effects in mice that manifest in bulk RNA sequencing (RNA-seq) as a biased expression of one parental allele over the other rather than the allele silencing typical of canonical imprinting (Bartolomei and Ferguson-Smith, 2011). We found evidence that noncanonical imprinting effects may reflect imprinted expression in discrete subpopulations of cells (Bonthuis et al., 2015). However, the functional significance and cellular nature of noncanonical imprinting remain largely unknown, and the total numbers of affected genes are debated. Targeted studies of individual genes are now essential to determine which genes have bona fide imprinting effects with important functional and ethological roles.

Among the genes affected by noncanonical imprinting in the adult brain, we found *dopa decarboxylase* (*Ddc*) and *tyrosine hydroxylase* (*Th*) (Bonthuis et al., 2015). *Th* is required for catecholamine biosynthesis, including dopamine (DA), norepinephrine (NE), and epinephrine (E), while *Ddc* is required for catecholamine and serotonin (5-HT) biosynthesis. Imprinting of *Ddc* was originally described as a transient developmental event in the embryonic heart (Menheniott et al., 2008). Functional roles for *Ddc* and *Th* imprinting are unknown but could affect behavior. One challenge to test this possibility is that the dissection of behavioral mechanisms that evolved under complex conditions in the wild is hampered by the lack of paradigms that emulate natural conditions to accurately reveal phenotypes (Gomez-Marin and Ghazanfar, 2019) and by a lack of technical and conceptual frameworks for decomposing and understanding the mechanistic basis of naturalistic behavior (Datta et al., 2019; Krakauer et al., 2017).

Monoaminergic systems are ancient, evolutionarily conserved mechanisms with important roles in decision making (Barron et al., 2010; Schultz et al., 2017) and in brain functions involved in foraging (Kamhi et al., 2017). DA, NE, E, and 5-HT affect feeding, reward, fear, anxiety, learning and memory, cognition, sensorimotor systems, and more (Gershman and Uchida, 2019; Klein et al., 2019; Okaty et al., 2019; Sara, 2009; Volkow et al., 2017). These neural systems help form foraging patterns that balance the costs of predation and energy expenditure with the benefits of caloric intake (Stephens, 2019). Increasing emphasis has been placed on studying foraging to identify mechanistic, evolutionary, and ethological rules governing mammalian decision making, cognition, and behavior (Gomez-Marin and Mainen, 2016; Gomez-Marin et al., 2014; Kalenscher and Wingerden, 2011; Pearson et al., 2014). Studies of foraging and decision making typically focus on binary choices (Hayden, 2018). However, in nature, the entire behavioral sequence before, during, and after a choice shapes the benefits and costs and is subject to natural selection. This prompted us to recently develop a different approach that uses unsupervised machine-learning methods to decompose naturalistic foraging patterns to improve our understanding of the mechanistic basis of the behavior in mice (Hörndli et al., 2019). We are now poised to test ethological roles for *Ddc* and *Th* imprinting in foraging.

Our naturalistic foraging paradigm mimics key natural elements, including a freely accessible home, food patches, and digging for seeds. To uncover naturalistic phenotypes, we measure human-defined keystone features of foraging, such as total food consumed and time in exposed regions of the environment. Next, we use an unsupervised machine-learning approach that detects, classifies, and counts discrete, reproducible foraging sequences from a large set of measures of gait, location, movement patterns reflecting different decisions, and more. Our machine-learning approach previously showed that naturalistic foraging is constructed from finite, genetically controlled behavioral sequences, which we call “modules” (Hörndli et al., 2019). Modules are reproducible behavioral sequences that range from less than a second to hundreds of seconds in duration. The expression of specific foraging modules in turn determines the values of specific keystone features of foraging outcomes. Our findings could be powerful for understanding imprinted gene functions in the brain and support proposals that top-down decompositions of a behavior, which first discover its organizational principles, are essential for understanding its mechanistic basis (Datta et al., 2019; Krakauer et al., 2017).

Homozygous ^−/−^ gene knockout mice reveal the phenotypes and biological processes that require the function of a particular gene. However, for heterozygous mutations, canonical imprinting impacts a mutation’s effects depending on the parental origin of the mutant allele (Peters, 2014). A phenotypic change may occur if the mutation resides in the expressed parental allele but not in the silenced allele. For noncanonical imprinting, the two alleles are differentially expressed. Loss of the function of one parental allele reveals the traits and biological process that require that allele. Thus, when compared with their ^+/+^ littermates, ^−/+^ mice with a null maternal allele reveal traits affected by that maternal allele. Conversely, reciprocal ^+/−^ mice with a null paternal allele reveal traits affected by the paternal allele. With this experimental design, we can uncover the maternal versus paternal allelic phenotypes for a given gene.

Here, we test the hypothesis that the maternal and paternal alleles for *Th* and *Ddc* have distinct roles in determining offspring foraging that are associated with differential allelic expression in subpopulations of cells and with differential effects on molecular and endocrine phenotypes. We test foraging in reciprocal *Th*^−/+^, *Th*^+/−^, *Ddc*^−/+^, *Ddc*^+^, and ^+/+^ littermate controls to uncover the maternal and paternal phenotypes for each gene in male and female adults. Further, functional interactions between *Th* and *Ddc* parental alleles are investigated using compound *Th*^−/+^*Ddc*^−/+^ and *Th*^+/−^*Ddc*^+/−^ reciprocal heterozygotes. For both *Th* and *Ddc,* our results show that the maternal and paternal alleles have distinct functional roles in foraging. Each allele affects the expression of specific decision and action sequences (i.e., modules). We create *Ddc*-allele-specific knockin reporter mice and study the cellular expression of the maternal versus paternal alleles across 52 adult brain regions and in different tissues. *Ddc* imprinting impacts allelic expression in specific subpopulations of brain and adrenal cells. Functional studies reveal different roles for *Ddc* parental alleles in regulating molecular expression profiles and endocrine components of the brain-adrenal axis. Overall, we show ethological, physiological, and molecular functions for noncanonical imprinting in the monoamine system and the brain-adrenal axis. Unique maternal and paternal allele phenotypes are revealed in sons and daughters, as well as foundations for imprinted gene models of foraging and decision control at the cellular level.

## RESULTS

### Identification of distinct ethological roles for *Th* and *Ddc* parental alleles in foraging behavior

**Figure 1.**
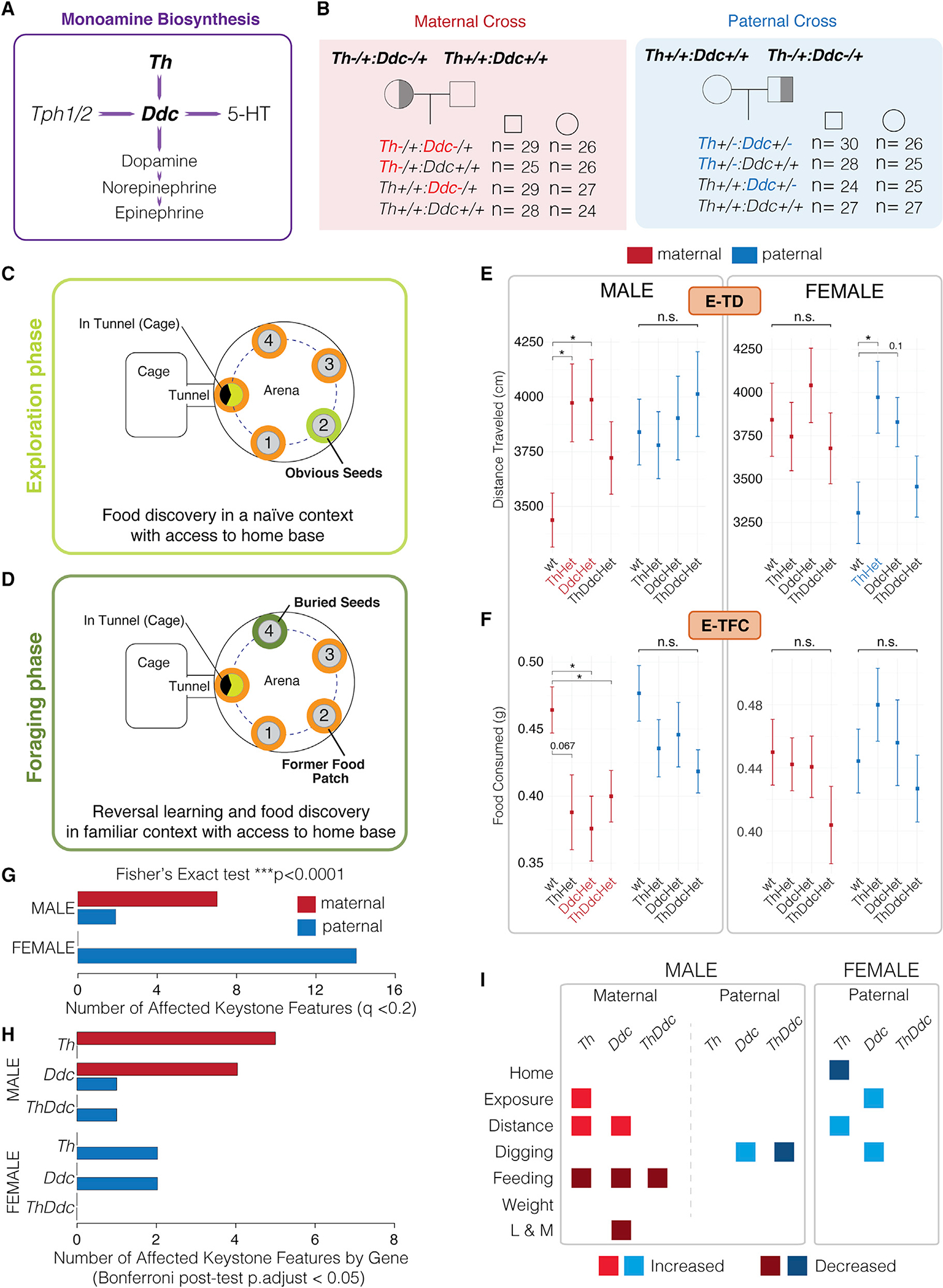
Maternal versus paternal *Th* and *Ddc* alleles affect distinct keystone features and foraging outcomes in males and females (A) Diagram of *Th* and *Ddc* within monoamine biosynthetic pathways. (B) Maternal (red) and paternal (blue) cross-breeding strategies for generating reciprocal *Th, Ddc*, and *ThDdc* heterozygous mice and littermate controls. Numbers of offspring tested are shown. (C and D) Schematics show the 30-min exploration (C) and foraging (D) phase tests to evaluate naive versus familiar context foraging, respectively. Exploration phase: seeds are placed on top of the sand in pot 2. Foraging phase: seeds are moved and buried in the sand in pot 4. Food-deprived mice forage for the seeds in each phase. (E and F) Plots show data for maternal (red) versus paternal (blue) mutant allele offspring for the total distance traveled in the exploration phase (E; E-TD) and the total food consumed (F; E-TFC) for males and females. Post-tests compare *Th, Ddc*, or compound *ThDdc* heterozygous mutant mice with wild-type (WT) littermates. Bonferroni-adjusted *p < 0.05* and **p < 0.01; mean ± SEM. (G) Barplots show the total significant foraging keystone features in males and females with mutant maternal versus paternal *Th* and/or *Ddc* alleles. The relative number of affected keystone features in each sex depends significantly on the parental origin of the allele (***p < 0.0001 Fisher’s exact test). See significant main effects in Figure S1. (H) Barplots showing the total number of significant keystone features for each allele and gene. Significant keystone features detected in *Th, Ddc*, or *ThDdc* heterozygous mice are relative to ^+/+^ littermates (Bonferroni-adjusted p < 0.05). See post-test effects in Figure S1. (I) A summary of how affected foraging keystone features impact foraging outcomes. Outcomes that are increased (bright red/blue) versus decreased (dark red/blue) are shown according to the significantly affected keystone features that are measures of these outcomes (see Figure S1).

We first determined how the maternal versus paternal *Th* and *Ddc* alleles affect offspring foraging behavior. Compound heterozygous males or females with null *Th* and *Ddc* alleles were bred with wild-type mice to generate *Th*, *Ddc*, and compound *ThDdc* heterozygous offspring (Figures 1A and 1B). We performed reciprocal crosses in which the null allele is maternally versus paternally derived (Figure 1B). Naturalistic foraging behaviors were then profiled using our published paradigm (Hörndli et al., 2019), in which the mouse’s home cage is attached to a foraging arena by a tunnel (Figures 1C and 1D). The mice forage spontaneously in two 30-min phases. During the exploration phase, naive mice explore the novel environment and discover and consume millet seeds in a food patch (Figure 1C, pot 2). Four hours later, the mice return to the arena in the foraging phase, in which the seeds are now buried in the sand in a new location (Figure 1D, pot 4). In this second 30-min phase, mice express behavior related to their expectation of food in the former food patch and the discovery of a new, hidden food source (pot 4). Mice express rich and diverse behavioral sequences in this paradigm (Video S1). We captured 426 h of foraging video footage from the 426 mice in the study to test for foraging phenotypes.

Our human-defined keystone features of foraging measure home-related behaviors, time in exposed regions of the arena, foraging distance traveled, digging, feeding, body weight, and learning- and memory-related behaviors (Figure S1, see sunflower plots; Table S1). Some keystone features are significantly and differentially affected by loss of the maternal versus paternal alleles (Figure S1). For example, males with a null maternal *Th* or *Ddc* allele display a significant increase in the total distance traveled relative to their wild-type littermates (Figure 1E, E-TD male, initial 30-min exploration phase). In contrast, males with a null paternal allele are not affected (Figure 1E). Furthermore, males with a null maternal *Th* and/or *Ddc* allele consume less food, while paternal mutants are not significantly affected (Figure 1F, E-TFC). The effects of each parental allele are also sex specific. While males with null maternal allele have significant changes to total distance traveled, we found the opposite parental pattern for females (Figure 1D, E-TD). Additionally, unlike males, a null maternal allele did not significantly change total food consumption in females (Figure 1F, E-TFC). These results show that loss of the maternal versus paternal *Th* and *Ddc* alleles affects foraging differently in sons and daughters.

We determined the total numbers of keystone features significantly affected by each parental allele for *Th* and/or *Ddc* according to sex and foraging phase (Figures S1C and S1F, see features labeled with an asterisk). We found that loss of the maternal *Th* and/or *Ddc* alleles affected 7 keystone features in males, while loss of paternal alleles affected 2 features (Figures 1G and S1B–S1F). In contrast, for females, only loss of the paternal allele caused significant effects, impacting 14 keystone features (Figures 1G and S1B–S1F). This difference in the maternal versus paternal phenotypes between males and females is statistically significant (p < 0.0001, Fisher’s exact test; Figure 1G). To compare *Th* and *Ddc,* which have different roles in monoamine synthesis, we performed post-tests on the significantly affected keystone features found above (Figures S1C–S1F, see features labeled with dark-colored bars). For both genes, maternal and paternal alleles significantly affected keystone features in males, while only paternal alleles affected females (Figure 1H). Maternal *Th* and *Ddc* alleles increased male exposure and distance traveled, decreased food consumption (feeding), and altered learning- and memory-related behavioral responses (Figures 1I and S1C). Paternal alleles changed male digging, including increasing digging in patches that did not contain food (pots 1 and 3) (Figures 1I and S1D). In females, some differences in the effects of *Th* and *Ddc* were apparent. Loss of the paternal *Th* allele decreased time in the home and increased the distance traveled (Figures 1I and S1F), while the paternal *Ddc* allele increased digging in a nonfood-containing patch and increased time in the exposed center of the foraging arena (Figures 1I and S1F). In conclusion, both *Th* and *Ddc* parental alleles show differential and sex-specific effects on foraging that involve a biased maternal influence on sons and a paternal influence on daughters.

Finally, we tested whether the phenotypic effects of maternal or paternal *Th* and *Ddc* mutant alleles are additive in compound *ThDdc* mutants and can increase the magnitude of *Th* and *Ddc* mutation effects (Figures 1E, 1F, and S1B–S1F). Unexpectedly, fewer keystone features are significantly affected in the compound mutants compared with the single-gene mutants (Figure 1H). To confirm this, we performed standard elevated zero maze, light-dark box, and open field tests of exploratory behavior (Figure S2). These standard tests support our findings by showing that *ThDdc* compound heterozogotes do not simply show additive increases in the magnitude of the effects caused by *Th* or *Ddc* alone but can even show suppression. For example, loss of the paternal *Th* allele significantly decreases the latency to exit the closed arm of the elevated zero maze in males, and the effect is unique to *Th* alone and suppressed in *ThDdc* compound heterozygotes (Figure S2A). Additionally, females with a null paternal *Ddc* allele show significantly increased time in the center during open field testing but not in *ThDdc* compound heterozygotes (Figure S2B), and phenotypic trends in a light-dark box test were not increased in *ThDdc* compound mutants (Figure S2C). Overall, *Th* and *Ddc* parental alleles do not additively affect a shared set of foraging components.

### Unsupervised machine learning reveals reproducible modules of foraging behavior

**Figure 2.**
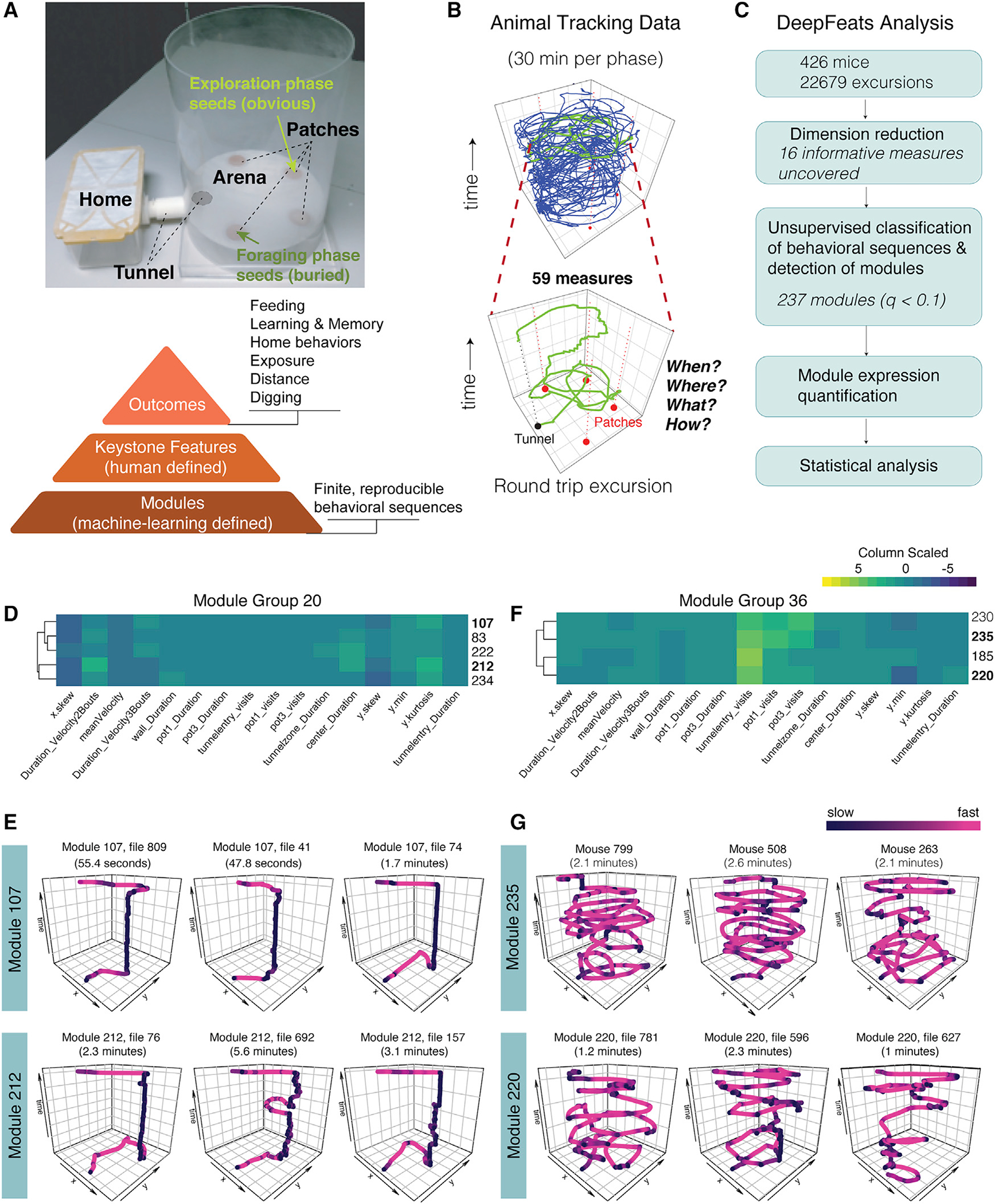
Unsupervised machine-learning analysis uncovers foraging modules (A) Naturalistic foraging task includes free access to the home and foraging patches of sand with seeds. Human-defined foraging keystone features measure what an animal does and outcomes of overt importance. Machine-learning-defined foraging modules reveal how an animal forages. (B) Trace of the foraging pattern of a single mouse during the exploration phase (blue track). The DeepFeats algorithm segments complex foraging into individual round-trip excursions from the home (green trace) and analyzes 59 different measures that describe what the animals do, where they go, how they move, and temporal patterns (see STAR Methods). (C) The DeepFeats analysis workflow for module discovery. The foraging patterns expressed by 426 adult male and female *Th*^−/+^, *Th*^+/−^, *Ddc*^−/+^, *Ddc*^+/−^, *ThDdc*^−/+^, *ThDdc*^+/−^, and WT littermates in the study revealed 237 distinct foraging modules from 22,679 round-trip foraging excursions (see Figure S3). (D and E) The heatmap (D) shows a subset of the total 237 foraging modules found (see Figure S4). The centroid describing the distinguishing measures of each module for a group of similar modules is shown (group 20: modules 107, 83, 222, 212, and 234) and the relative magnitude of the 16 measures delineating different modules. (E) Traces of three representative foraging sequences from different mice for module 107 and 212 types are shown. Movement velocity is indicated by the color (see legend). (F and G) Heatmap data for modules from group 36 (F) and representative traces for foraging sequences of module 235 and 220 types are shown.

We sought to learn how maternal and paternal alleles contribute to different foraging outcomes. A mechanism could affect how a mouse behaviorally extracts and eats food (module level, machine learning) without changing how much total food is consumed (keystone feature level, human defined). Here, we test the hypothesis that the maternal versus paternal *Th* and *Ddc* alleles cause different foraging outcomes by affecting finite decision and action sequences that we call foraging modules (Hörndli et al., 2019) (Figure 2A). We began by determining the identity of the modules expressed by decomposing the mouse foraging patterns using our DeepFeats algorithm (see STAR Methods). For each round-trip excursion from the home, we captured measures of gait, locations visited, latencies, timing, and more. Following dimension reduction, unsupervised machine learning revealed significantly reproducible foraging sequences indicative of modules (Figures 2B and 2C). The male and female mice profiled expressed a total of 22,679 round-trip foraging excursions, and DeepFeats identified 16 measures that best detect candidate modules (Figures 2C, S3A, and S3B). Our training dataset partition revealed clusters of similar foraging sequences, which were tested for significant reproducibility in our test set partition (Figures S3C and S3D). This found 237 significant foraging modules (Figures 2C, S3C, and S3D), and we next evaluated their validity.

Each module was assigned an ID number, and centroids were computed from the clustered data for each module, yielding behavioral markers for each module. Unsupervised hierarchical clustering of the module centroids revealed groups of similar modules (Figure S4). DeepFeats visualization tools confirmed that different modules involve different behavioral sequences, as shown by representative traces of the movement and decision sequences (Figure S4). In one example, modules 107 and 212 are in centroid group 20 (Figures 2D, 2E, and S4), while modules 235 and 220 are in group 36 (Figures 2F, 2G, and S4). These data show that foraging sequences assigned to the same module are similar, as expected (Figures 2E and 2G). Moreover, foraging modules within a group, such as modules 107 and 212 (Figure 2E), are more similar than modules from a different group, such as modules 235 and 220 (Figure 2G). Heatmaps of the centroid data revealed the measures that differentiate modules and module groups (Figures 2D, 2F, and S4), such as differences in the minimum movement in the y plane (y.min), visits to pots 1 and 3, duration near the wall, mean velocity, and more (Figures 2D, 2F, and S4). Video examples of the module behavioral sequences are shown in Video S2.

To initially evaluate biological validity, we tested whether the modules capture changes to foraging patterns over time, which would not occur for random noise. Moreover, we tested whether they are sensitive to sex differences and, therefore, biological effects. Transitions between different modules were defined for males (Figure S4B) and females (Figure S4C), and we found that the expression of a given module depends significantly on the identity of the previously expressed module. Thus, modules are expressed in a stereotyped sequence and capture foraging changes over time. We compared male versus female module transition matrices by contrasting the Euclidean distance of the stationary probability distribution for each matrix using a permutation test. A significant difference in male versus female module expression sequences was found (Figures S4B and S4C). Thus, we uncovered biologically valid foraging modules that capture behavior changes over time and biological differences. Building on this, we next determined how maternal versus paternal alleles shape foraging outcomes by affecting modules.

### Maternal versus paternal *Th* and *Ddc* alleles control the expression of distinct modules affecting foraging outcomes in males and females

**Figure 3.**
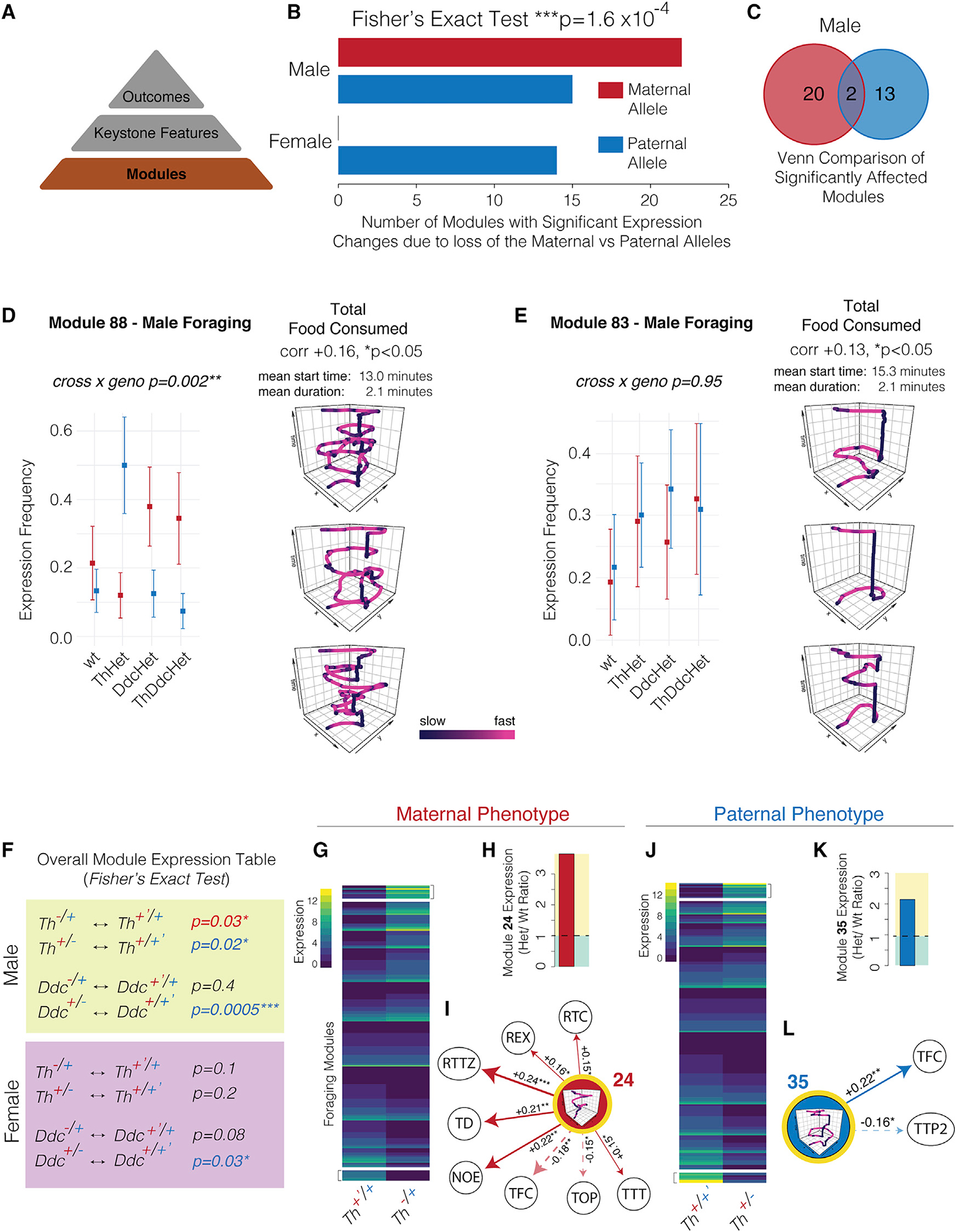
Maternal and paternal *Th* and *Ddc* alleles affect the expression of distinct subsets of foraging modules in males, while only paternal alleles affect females (A and B) An analysis of foraging modules (A). The barplot (B) shows the numbers of modules in male or female mice that are significantly affected by loss of the maternal (red) versus paternal (blue) *Th* and/or *Ddc* alleles (q < 0.2, generalized linear regression main effect of genotype). The relative numbers of affected modules in males versus females depends significantly on the parental origin of the mutant allele (p < 0.0001, Fisher’s exact test). (C) The Venn diagram compares the significantly affected modules in males due to loss of the maternal (red) versus paternal (blue) alleles. (D) The plot shows the foraging phase expression for a module (module 88) that is significantly affected by the parental origin of the mutant allele in males (**p = 0.002, generalized linear model, parent × genotype interaction, n = 24–30). The expression of the module in maternal (red) versus paternal (blue) allele mutants differs for *Th, Ddc*, and *ThDdc* heterozygotes. The traces show examples of module 88 foraging sequences from 3 different mice along with the mean expression start time and duration. The expression of this module is significantly associated with increased total food consumption (*p < 0.05, linear model; Corr, Pearson’s r) and shows complex exploratory behavior with intervening stops at the food patch (pot 4). Mean ± SEM. (E) The plot shows module 83 expression for males in the foraging phase. Module 83 expression is not significantly affected by loss of the maternal versus paternal alleles (n = 24–30) yet is also a significant predictor of more total food consumed, like module 88. Traces show that module 83 is a more directed sequence orientated at the food patch (pot 4) compared with module 88. (F) The table shows the results of a Fisher’s exact test performed on a count table of the expression frequencies of each of 237 modules for the indicated genotype and parental cross-contrasts. In males, loss of the maternal and paternal *Th* alleles and the paternal *Ddc* allele significantly changes module expression. For females, loss of the paternal *Ddc* allele changed module expression (n = 24–30 mice). (G–I) Specific modules are affected by loss of the maternal *Th* allele, and each affected module impacts specific keystone features. A heatmap (G) of the foraging phase module expression count table (y axis shows 237 modules) compares male mice with a null maternal *Th* allele to their ^+/+^ littermates. Subsets of modules with relatively increased or decreased expression are indicated. The plot in (H) shows aggregated count data for module 24, which exhibits increased expression in *Th*^−/+^ compared with ^+/+^ mice. (I) Module 24 expression (representative foraging trace shown) is significantly associated with increases (solid arrow) and decreases (dashed arrow) in specific keystone features (linear model, *p < 0.05, **p < 0.01, ***p < 0.001; n = 426 mice; Pearson’s r value shown). TFC, total food consumed; RTC, relative time in center; REX, relative exploration; RTTZ, relative time in tunnel zone to home; TD, total distance; NOE, number of excursions; TOP, time on the arena platform; TTT, total time in tunnel to home (see Table S1). (J–L) Loss of the paternal *Th* allele affects different modules compared with the maternal allele. The heatmap (J) of the foraging phase module expression count table (y axis shows 237 modules) compares male mice with a null paternal *Th* allele to their ^+/+^ littermates. The plot in (K) shows aggregated count data for module 35, which exhibits increased expression in paternal allele mutant *Th*^+/−^ compared with ^+/+^ mice. (L) Module 35 expression (representative foraging trace shown) is significantly associated with increased TFC and decreased total time at pot 2 (TTP2), the former food patch.

We determined whether the maternal versus paternal *Th* and *Ddc* alleles differentially affect the expression frequencies of specific foraging modules. In males, we found that 22 modules are significantly affected by the loss of maternal *Th* and/or *Ddc* alleles and that 15 are affected by the paternal alleles (Figure 3B). The maternally versus paternally affected modules differ, showing that each allele significantly affects different modules (Figure 3C). In females, only the paternal allele significantly affected module expression, impacting 14 of the 191 modules tested after filtering for modules with low-expression variance (Figure 3B). Like the keystone features above, the numbers of significantly affected modules in males versus females depend significantly on the parental origin of the null allele (Figure 3B). Maternal influence predominates in males compared with paternal influence in females. Thus, module-level effects parallel the keystone-feature-level effects. Linear modeling uncovered the identities of the modules that are statistically significant predictors for increases or decreases in the values for each keystone feature (Figure S5). By linking modules to keystone features, we were able to next determine how allelic effects on module expression predict changes to keystone features of foraging outcomes.

In one example, we found that module 88 has a significant parent-of-origin allelic effect on expression in males. Increased expression occurs with a null paternal *Th* allele, but increased expression is associated with a null maternal allele for *Ddc* and *ThDdc* (Figure 3D). In an informative contrast, we found that a related module, module 83, is not significantly affected (Figure 3E). However, both modules 88 and 83 are significant predictors of increased total food consumption in males (Figures 3D, 3E, inset, and S5), and both modules are expressed during the foraging phase, yet they show key differences in how the mice exploit food. Module 88 behavioral sequences are ~2 min in duration, expressed 13 min into testing, and include two extended stops at the food patch (pot 4) interrupted by looping explorations of the environment and several gait changes (Figure 3D). However, module 83 is also ~2 min in length and is typically expressed 15 min into testing but, unlike module 88, involves extended visits to the food patch with few looping explorations and relatively decreased gait complexity (Figure 3E). These findings show that the maternal and paternal *Th* and *Ddc* alleles differentially impact discrete foraging action and decision sequences (modules) linked to particular foraging outcomes, such as total food consumption in this example.

We determined the full profile of affected modules for each parental allele and gene. The expression of each module was tallied for the heterozygous and wild-type mice to create a module expression count matrix (237 modules [rows] by 2 genotypes [columns]), and a Fisher’s exact test revealed whether the relative expression frequency of the modules depends significantly on the allelic genotype of the offspring. Both maternal (^−/+^) and paternal (^+/−^) *Th* alleles significantly affect module expression profiles in males compared with ^+/+^ littermate controls, and the paternal *Ddc* allele (^+/−^) significantly affects module expression profiles in both males and females (Figure 3F). We were initially surprised by the absence of a significant effect for the maternal *Ddc* allele (Figure 3F), which appeared inconsistent with our keystone feature results. However, a post-test analysis found the expected trend for a maternal *Ddc* allele effect in the foraging phase data (p = 0.07, Fisher’s exact test). Next, unsupervised hierarchical clustering revealed the most strongly affected modules for each allele and gene, and our linear modeling analysis revealed how these modules link to the keystone features (Figure S5). For example, loss of the maternal versus paternal *Th* alleles causes different modules to change their expression (Figures 3G and 3J). Module 24 expression increases with loss of the maternal allele (Figure 3H) and, in turn, is significantly associated with increases in 6 keystone features of exploratory behavior and with decreases in 2 features of feeding (Figure 3I). On the other hand, module 35 expression increases following loss of the paternal allele (Figure 3K), and this module promotes increased total food consumption and decreased total time at the former food patch (pot 2) in the foraging phase (Figure 3L). Therefore, maternal and paternal alleles determine keystone features of foraging outcomes by, at least in part, affecting the expression of specific modules.

### Allelic reporter mice reveal discrete cell populations exhibiting dominant expression of the maternal *Ddc* allele in 14 adult brain regions

**Figure 4.**
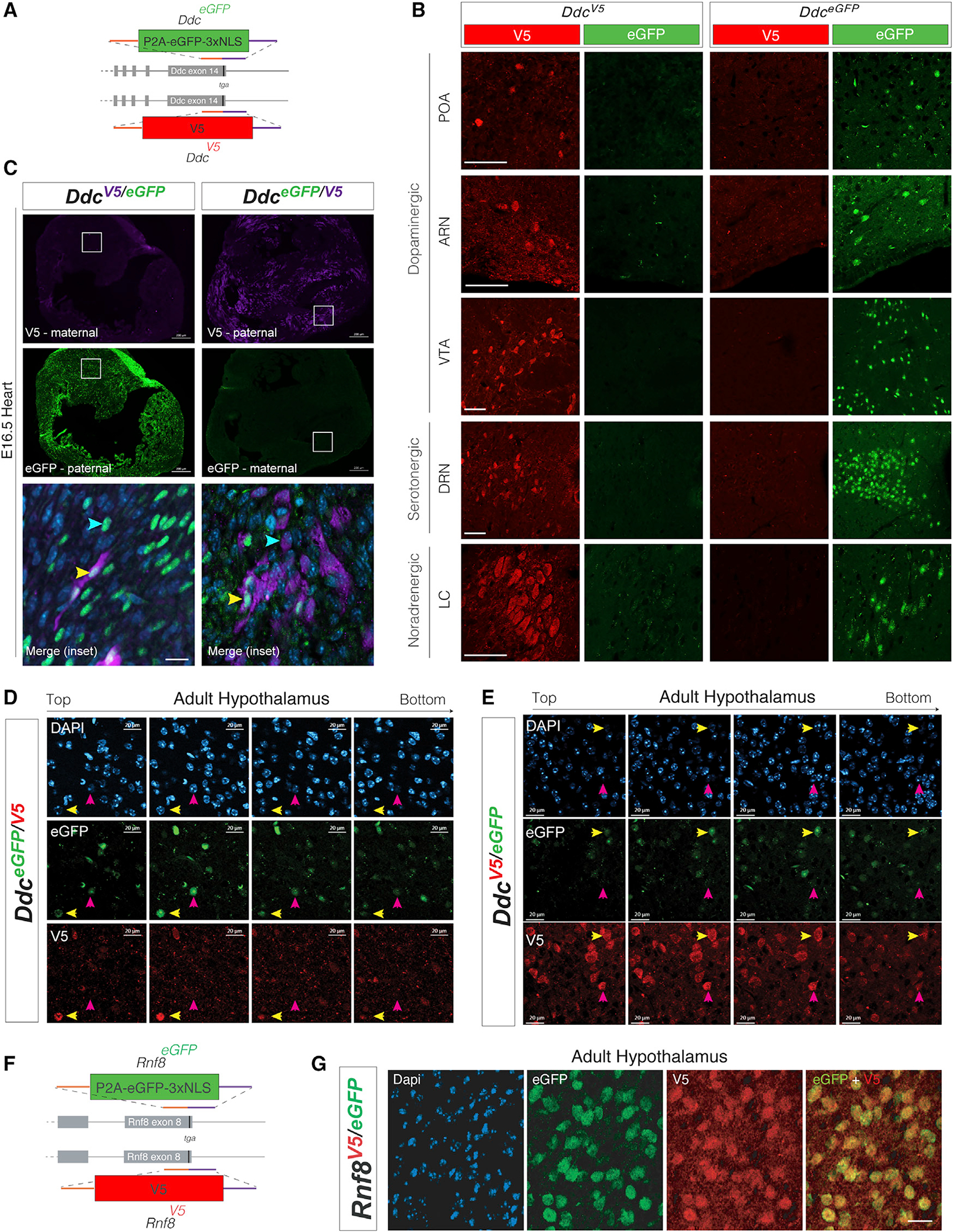
Knockin reporter mice reveal *Ddc* allelic expression at the cellular level (A) Schematic summary of *Ddc* allelic knockin reporter mouse design. (B) Images show expression of the V5-tagged DDC protein in *Ddc*^*V5*^ mice and nuclear EGFP protein expression in *Ddc*^*EGFP*^ reporter mice for major monoaminergic brain nuclei in the adult brain. POA, preoptic area; ARN, arcuate nucleus; VTA, ventral tegmental area; DRN, dorsal raphe nucleus; LC, locus coeruleus. Size bars are 50 μm. (C) Images of compound *Ddc*^*EGFP/V5*^ and *Ddc*^*V5/EGFP*^ allelic reporter mice reveal preferential paternal allele expression (blue arrows) in the developing embryonic day 16.5 (E16.5) heart. DDC+ myocardial cells that express both parental alleles are also revealed (yellow arrows). Size bar is 10 μm. (D and E) Optical sections of *Ddc*^*EGFP/V5*^ (D) and *Ddc*^*V5/EGFP*^ (E) adult female hypothalamus reveal subpopulations of neurons expressing both *Ddc* alleles equally (yellow arrows) and subpopulations with maternal allele expression (pink arrows). z stack shown. Size bars are 20 μm. (F and G) Control allelic reporter mice generated for *Rnf8,* a gene that is not imprinted (F), show co-expression of both parental alleles in all expressing hypothalamic cells (G), as expected.

*Th* and *Ddc* imprinting effects manifest as an allele bias in bulk RNA-seq data but could in fact be preferential expression of one allele in a specific subpopulation of cells and not others (Bonthuis et al., 2015). To test this and begin to dissect the mechanistic basis of the foraging phenotypes, we created two *Ddc* knockin reporter mouse lines. One reporter line has a C-terminal V5 epitope tag attached to the DDC protein for direct detection of one allele (Figure 4A). A second line contains a stable, nuclear EGFP reporter separated from DDC by a self-cleaving P2A peptide sequence (Figure 4A). Our *Ddc*^*EGFP*^ line produced robust nuclear EGFP expression in cells for all major monoaminergic brain regions (Figure 4B). Similarly, the DDC is directly detectable in *Ddc*^*V5*^-targeted knockin mice using immunolabeling with an anti-V5 protein tag antibody (Figure 4B). To compare the cellular expression of the maternal versus paternal *Ddc* alleles, we generated reciprocal *Ddc*^*EGFP*/*V5*^ and *Ddc*^*V5/EGFP*^ allelic reporter mice. Since EGFP is stable with a half-life greater than 26 h (Corish and Tyler-Smith, 1999), we can infer that any V5+ and EGFP− monoallelic brain cells had stable silencing of the EGFP allele for days, at least. The embryonic day 16 (E16) heart shows transient preferential paternal *Ddc* allele expression (Menheniott et al., 2008). As expected, we found expression of the paternal allele and silencing of the maternal allele in E16 cardiac tissue from *Ddc*^*EGFP*/*V5*^ and from *Ddc*^*V5/EGFP*^ mice (Figure 4C). We also uncovered a small subpopulation of heart cells that express both parental alleles (Figure 4C, yellow arrow), indicating cell-specific *Ddc* imprinting effects. Thus, our reporter lines perform as expected, and we began a detailed study of the adult brain.

In *Ddc*^*EGFP/V5*^ mice, optical sectioning of the hypothalamus revealed that most DDC+ cells express both parental alleles (Figures 4D and 4E, yellow arrows); however, a subpopulation exhibit preferential maternal allele expression in *Ddc*^*EGFP*/*V5*^ and *Ddc*^*V5/EGFP*^ reporter lines (Figures 4D and 4E, pink arrows). This finding was observed in males and females and is consistent with the maternal allele bias observed in bulk hypothalamic RNA-seq data (Bonthuis et al., 2015). We did not observe any cells with preferential paternal allele expression. In a control study, we generated allelic reporter mice for *Rnf8,* a gene that does not exhibit imprinting effects (Bonthuis et al., 2015). As expected, all RNF8+ cells in the hypothalamus (and other regions) co-express both parental alleles, and cellular imprinting effects were not observed (Figures 4F and 4G). We next created a brain-wide map of *Ddc* cellular imprinting effects.

**Figure 5.**
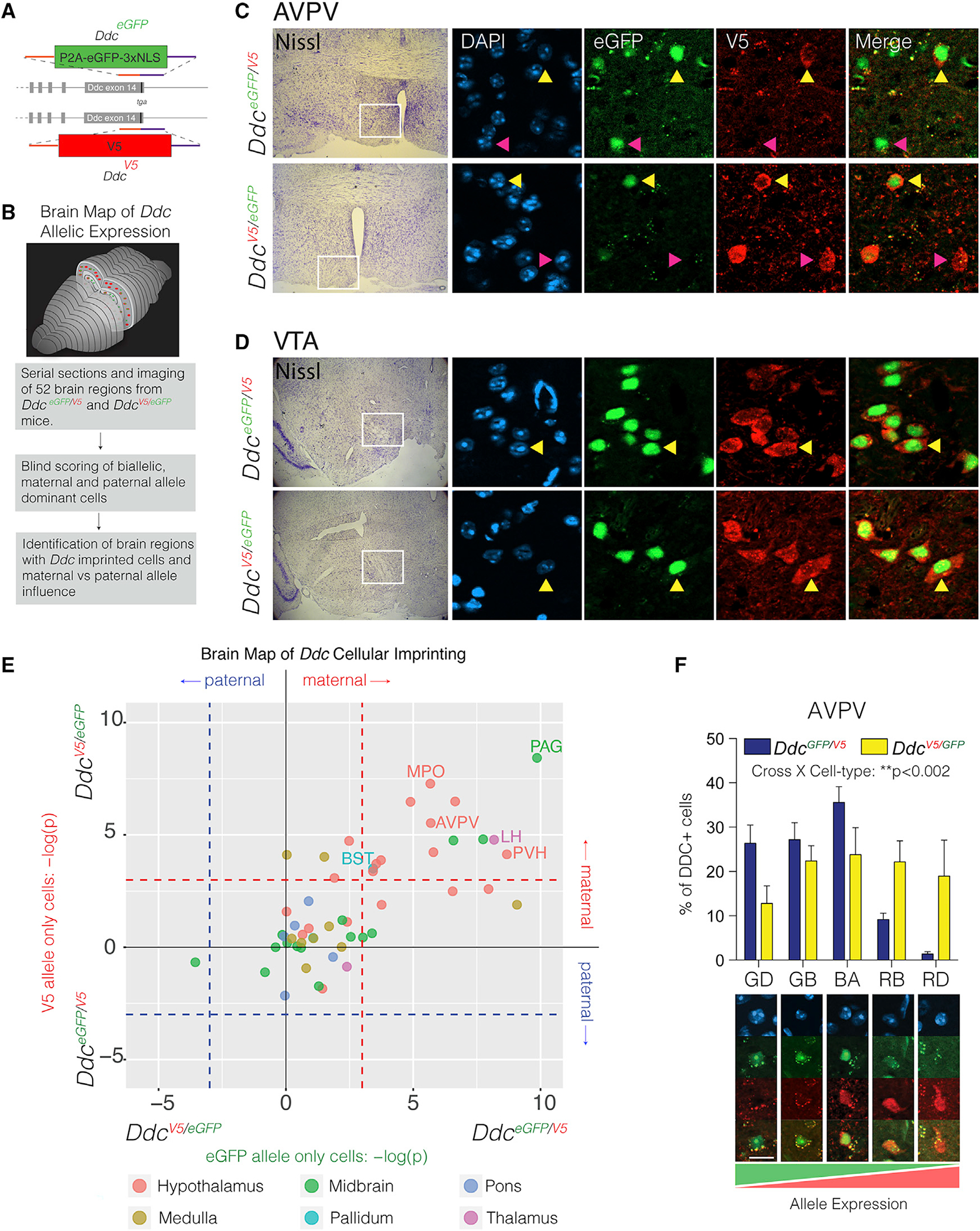
Brain-wide analysis identifies 14 adult mouse brain regions containing DDC+ neurons with preferential maternal allele expression (A and B) Schematics showing the generation (A) and brain-wide analysis (B) of reciprocal *Ddc* allelic reporter mice to uncover brain regions with DDC+ cells exhibiting imprinting effects. (C and D) Images show maternal-allele-expressing neurons in the AVPV of the hypothalamus (pink arrow) along with neurons expressing both alleles (yellow arrow) (C). In the VTA, only neurons expressing both alleles are observed (D). The white box in the Nissl-labeled section shows the location of the imaged brain region. (E) The scatterplot shows the results of an analysis of the presence versus absence of maternal- or paternal-allele-expressing cells in 52 brain regions for reciprocal *Ddc*^*EGFP/V5*^ and *Ddc*^*V5/EGFP*^ mice. Only brain regions harboring maternal-allele-expressing cells were observed (upper-right quandrant) and most impacted regions are in the hypothalamus (see legend). A Fisher’s test of a contingency table of the scored images determined significant maternal- or paternal-allele-expressing cells (p < 0.05, see STAR Methods; n = 2 per cross). AVPV, anteroventral periventricular nucleus; BST, basal nucleus stria terminalis; LH, lateral hypothalamic nucleus; MPO, medial preoptic area; PAG, periaqueductal gray; PVH, periventricular nucleus of the hypothalamus. See full atlas in Data S2. (F) The barplot shows quantification of cell allele expression in the AVPV in reciprocal *Ddc*^*EGFP/V5*^ and *Ddc*^*V5/EGFP*^ mice. The percentage of DDC+ cells that are EGFP+ dominant (GD), EGFP+ biased (GB), equal biallelic (BA), red V5+ biased (RB), and red V5+ dominant (RD) are shown. Examples of each cell category are shown below. Two-way ANOVA found a significant interaction between cross and allelic cell types (p < 0.002, n = 5). Mean ± SEM.

We tested for the presence versus absence of cells with preferential maternal or paternal *Ddc* allele expression for 52 different brain regions in *Ddc*^*EGFP/V5*^ and *Ddc*^*V5/EGFP*^ adult female mice (Figures 5A and 5B). Every fluorescence image was compared with an adjacent, parallel Nissl-stained section to define the anatomical location of the brain region(s) according to the Allen Brain Reference Atlas. The analysis uncovered brain regions with cellular imprinting effects (Figure 5C, anteroventral periventricular nucleus [AVPV], pink arrows) and regions without (Figure 5D, ventral tegmental area [VTA], yellow arrows). To test whether a brain region is significant for maternal- or paternal-allele-expressing cells, the number of images with EGFP or V5 allele-exclusive cells was statistically compared between the two crosses (*Ddc*^*EGFP/V5*^ and *Ddc*^*V5EeGFP*^) using a Fisher’s exact test (Figure 5E). We uncovered 14 brain regions significantly enriched for cells with dominant maternal allele expression confirmed in both crosses (Figure 5E, upper-right quadrant), while 38 brain regions contain DDC+ cells without overt imprinting effects (Figure 5E). Of the 14 brain regions with maternal-allele-expressing cells, 9 are in the hypothalamus (64%), one is in the pallidum, one is in the thalamus, and three reside in the midbrain. We did not identify any brain regions with paternal-allele-only-expressing cells (full atlas in Data S1 and brain region defined in Table S2).

For validation, we performed an independent and quantitative assessment of allelic expression in DDC+ neurons of the AVPV, a top region enriched for maternal-allele-expressing cells (Figure 5E). DDC+ cells were blindly scored into one of five allelic categories in the reporter mice: EGFP only, EGFP biased, biallelic, V5 biased, or V5 only (Figure 5F). We found a significant interaction between cross (*Ddc*^*EGFP/V5*^ and *Ddc*^*V5/EGFP*^) and genotype (Figure 5F), such that a shift toward EGFP+ allelic expression occurs in the *Ddc*^*EGFP/V5*^ mice, and a reciprocal distribution shift toward Ddc-V5 allelic expression occurs in the *Ddc*^*V5/EGFP*^ mice (Figure 5F). Thus, we confirmed preferential expression of the maternal *Ddc* allele in subsets of AVPV cells. Some EGFP+ monoallelic cells were observed in the *Ddc*^*V5/EGFP*^ cross, suggesting paternal allele expression, but they were not observed in the reciprocal *Ddc*^*EGFP/V5*^ mice and are therefore not bona fide imprinting effects. Overall, *Ddc* imprinting effects in the adult brain involve preferential maternal allele expression in subsets of cells in specific brain regions.

### *Ddc* imprinting effects impact discrete subpopulations of adrenal cells and determine brain-adrenal axis functions

**Figure 6.**
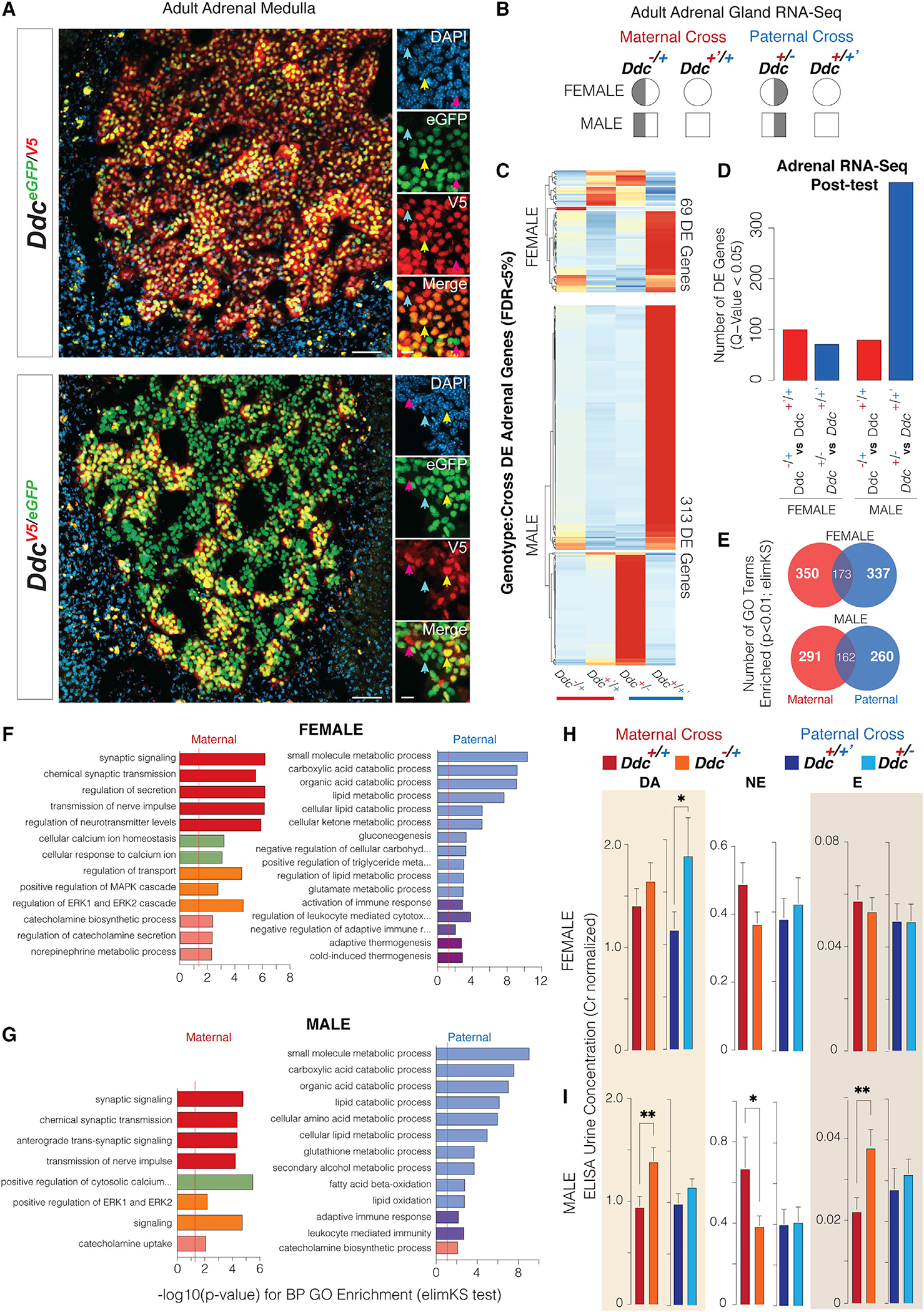
*Ddc* imprinting reveals parental controls over subsets of adrenal cells and specific molecular pathways and brain-adrenal endocrine outputs (A) The images show mixed DDC+ cell populations with paternal allele (blue arrows), maternal allele (pink arrows), and biallelic expression (yellow arrows) in the adrenal medulla. Low-magnification images of *Ddc*^*EGFP/V5*^ and *Ddc*^*V5/EGFP*^ cells show groups of biallelic cells and many paternal-allele-expressing cells (size bar is 100 μm). Insets show high-magnification images of cells with different allelic expression effects. Size bar is 20 μm. (B–E) The schematic (B) shows the experimental design for RNA-seq profiling to uncover molecular pathways differentially controlled by maternal versus paternal *Ddc* alleles in the female and male adrenal gland (n = 4). The heatmap (C) shows the RNA-seq differential expression results for genes with a significant interaction effect between genotype and parental crosses (generalized linear model, DESeq2, false discovery rate [FDR] < 5%). The barplot (D) shows the results of a post-test revealing the number of genes with significant differential expression from comparisons of maternal (red) or paternal (blue) *Ddc* allelic mutants with ^+/+^ littermates (t test post-test, DESeq2, FDR < 5%). The Venn diagrams in (E) compare the significant GO terms found for differentially expressed genes in *Ddc* maternal (red) versus paternal (blue) allelic mutant females and males. (F and G) The barplots show the top Gene Ontology biological process enrichments for the female (F) and male (G) differentially expressed gene sets in (D) (TOPGO elimKS test, p < 0.01). See Table S4. (H and I) The barplots show ELISA-detected levels of DA, NE, and E in the urine of female (H) and male (I) mice with null maternal (red) or paternal (blue) *Ddc* alleles compared with ^+/+^ littermates (creatinine-normalized values shown, t test, n = 20). *p < 0.05, **p < 0.01.

In addition to the brain, the adrenal medulla is a major site of catecholamine synthesis, and changes to adrenal *Ddc* expression could affect behavior and physiology (Eiden and Jiang, 2018). We tested for *Ddc* imprinting effects in the adrenal medulla with our allelic reporter mice and found that a major subpopulation of DDC+ adrenal cells preferentially expresses the paternal allele (Figure 6A, blue arrows), a minor subset preferentially expresses the maternal allele (Figure 6A, pink arrows), and a third subpopulation expresses both parental alleles equally (yellow arrows). These unique subpopulations of adrenal cells were confirmed in reciprocal *Ddc*^*EGFP*/*V5*^ and *Ddc*^*V5/EGFP*^ allelic reporter mice (Figure 5H). These findings suggest that *Ddc* imprinting effects mediate parental controls over brain-adrenal axis functions in offspring, which we tested next.

To test whether the maternal versus paternal *Ddc* alleles have different functional roles at the molecular level in the adrenal gland, we performed RNA-seq gene expression profiling on adrenal glands dissected from reciprocal *Ddc*^−/+^ and *Ddc*^+−-^ mice and their *Ddc*^+/+^ littermates (Figure 6B). Loss of the paternal allele caused a larger decrease in *Ddc* expression compared with the maternal allele (Figure S6A), consistent with more cells preferentially expressing the paternal allele, as observed in our reporter mice above. We then fit a model testing for an interaction between a genotype and parental cross to define imprinting effects on adrenal gene expression. We found 69 significantly affected genes in females and 313 in males (Figure 6C). Post-tests determined which genes are affected by loss of the maternal versus paternal *Ddc* alleles in males and females (Figure 6D; Table S3). Gene Ontology enrichment analysis of these genes relative to all genes expressed in the adrenals revealed that the maternal and paternal alleles largely effect different adrenal biological processes (Figure 6E; Table S4). Loss of the maternal allele most significantly affected neural-related processes, including synaptic functions in the adrenal medulla, as well as ERK and catecholamine signaling (Figures 6F and 6G, maternal). In contrast, loss of the paternal allele predominantly affected adrenal metabolic processes, including carboxylic acid, lipid and ketone metabolism, and thermogenesis, as well as some immune-related pathways (Figures 6F and 6G, paternal). Other important biological processes are also significantly affected (Table S4). Overall, the results show that the maternal and paternal *Ddc* alleles affect distinct molecular processes in the adrenal gland.

We next tested whether *Ddc* imprinting effects impact brain-adrenal axis catecholamine outputs, which can be detected in urine. We analyzed males and females separately because we found sex differences in adrenal gene expression (Figure S7B), and ELISA measured levels of urine DA, NE, and E levels (Figure S7C). Our study found that loss of the paternal *Ddc* allele in females caused significantly increased DA levels, while significant changes due to a loss of the maternal allele were not observed (Figure 6H). The opposite occurred in males, for which loss of the maternal *Ddc* allele caused significantly increased DA, decreased NE, and increased E, and loss of the paternal allele did not have significant effects (Figure 6I). Thus, *Ddc* parental alleles play distinct roles in regulating adrenal molecular processes and catecholamine outputs. Maternal allele effects on catecholamine outputs in sons and paternal allele effects on daughters parallel the foraging phenotype.

### A framework for imprinted gene cellular controls over the monoamine system

**Figure 7.**
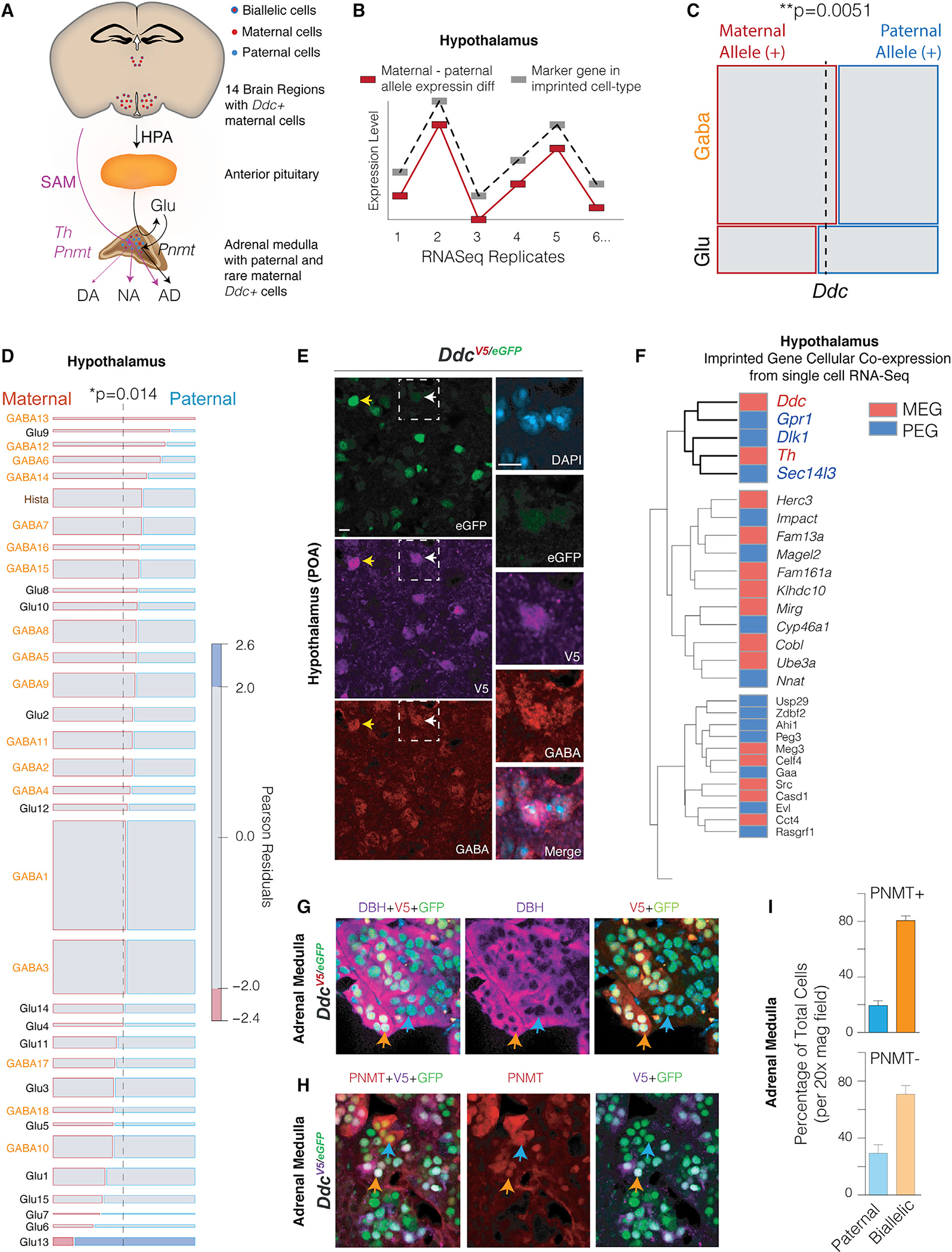
Identification of imprinted gene controls in molecular subtypes of monoaminergic hypothalamic and adrenal cells (A) The schematic summarizes the sympathetic-adrenal-medullary (SAM) and hypothalamic-pituitary-adrenal (HPA) components of the brain-adrenal axis and *Ddc* allelic expression. (B) The schematic graph shows the logic of the Imprinting-Expression Network (IEN) analysis that matches variance in cell-type marker gene expression to the magnitude of *Ddc* imprinting effects across bulk RNA-seq replicates to reveal major cell types with imprinting effects. (C) The mosaic plot shows the results of an IEN analysis for linkage of *Ddc* hypothalamic imprinting effects to the expression of GABAergic (Gaba) versus glutamatergic (Glu) neuron marker genes. *Ddc* imprinting effects are significantly dependent on neuron class (**p = 0.005, chi-square test), and maternal allele expression is associated with GABA neurons (dashed black line indicates the null hypothesis). (D) The mosaic plot shows the results of an IEN analysis for linkage of *Ddc* imprinting effects to gene markers of hypothalamic neuron subtypes from published single-cell RNA-seq. *Ddc* imprinting effects depend significantly on cell type (*p = 0.01, chi-square test). GABA subtypes (orange text) are more associated with maternal allele expression than Glu types (black text). (E) Co-immunolabeling of GABA, EGFP, and V5 in *Ddc*^*V5/EGFP*^ preoptic area confirms that subsets of hypothalamic GABAergic neurons exhibit maternal *Ddc* allele expression (white arrow, V5 expression, box indicates magnified cell in right-side images). Other GABA+ cells express both alleles (yellow arrow). Size bars are 10 μm. (F) Unsupervised hierarchical clustering of hypothalamus single-cell RNA-seq (scRNA-seq) data from 17,000 cells shows sets of co-expressed MEGs (red) and PEGs (blue). A set of imprinted genes co-expressed with *Ddc* reveal parental controls over monoaminergic cells. See Figure S7 for full data. (G and H) Immuno-labeling of DBH (G) and PNMT (H) in the adrenal medulla of *Ddc*^*V5/EGFP*^ mice reveals subsets of NE- and E-synthesizing cells, respectively, that exhibit paternal allele (blue arrow) versus biallelic expression (orange arrow). All DDC+ cells express DBH. (I) The bar plot shows the percentage of PNMT+ (dark bars) and PNMT− (light bars) adrenal cells that exhibit paternal *Ddc* allele expression (blue bar) versus expression of both alleles (orange bar).

Our above results show cellular roles for monoaminergic system noncanonical imprinting effects in foraging and brain-adrenal axis functions (Figure 7A). Our final aim was to create foundations for future work by defining candidate cell types and other imprinted genes involved. In the brain, some monoaminergic neurons are glutamatergic, and others are GABAergic (Trudeau and Mestikawy, 2018). To test whether maternal *Ddc* allele expression is preferentially linked to glutamatergic versus GABAergic neuronal identity in the hypothalamus, we devised a method based on gene co-expression analyses to uncover molecular subtypes of brain cells from bulk RNA-seq (Kelley and Berridge, 2002; Parikshak et al., 2013; Willsey et al., 2013). Our approach tests whether the magnitude of the imprinting effect for *Ddc* correlates with the expression level of glutamatergic versus GABAergic neuronal marker genes in bulk RNA-seq replicates (Figure 7B). A *Ddc* Imprinting ~ Cell Marker Expression correlation network (IEN) was generated using our published bulk RNA-seq replicates for the hypothalamus from adult female F1cb (CastEiJ × C57BL/6J, n = 9) and F1bc (C57BL/6J × CastEiJ, n = 9) hybrid mice (Bonthuis et al., 2015). We used marker genes previously found for 18 different GABAergic and 15 glutamatergic hypothalamic neuron types (Chen et al., 2017). We found that the relative expression of the maternal versus paternal *Ddc* alleles depends significantly on GABA versus glutamate neuron marker expression (Figure 7C). Maternal *Ddc* allele expression is positively associated with GABAergic markers, indicating preferential maternal allele expression in GABA neurons (Figure 7C).

By focusing on marker genes for subtypes of glutamatergic and GABAergic neurons defined from the single-cell RNA-seq work of Chen et al. (2017), we further found that relative maternal versus paternal *Ddc* allele expression is significantly dependent on specific neuronal subtypes (Figure 7D, p = 0.014, chi-square test). The majority (71%) of neuron types positively associated with maternal *Ddc* allele expression are GABAergic (Figure 7D), of which GABA13 and GABA12 neuron markers are most strongly linked. Immunohistochemical triple labeling of GABA, EGFP, and DDC-V5 in *Ddc*^*V5/EGFP*^ transgenic mice confirmed that hypothalamic cells exhibiting dominant expression of the maternal *Ddc* allele co-express GABA (Figure 7E, white arrows and zoomed images). Expression of GABA is not unique to the imprinted DDC+ neurons since GABA co-expression was also observed in biallelic DDC+ cells (Figure 7E, yellow arrows). In conclusion, *Ddc* hypothalamic imprinting effects are enriched in subtypes of GABAergic neurons.

Using published single-cell RNA-seq data (Chen et al., 2017), we next uncovered imprinted genes that are co-expressed with *Ddc* at the cellular level in the hypothalamus. With data from over 17,000 cells, we computed the mean expression levels of different imprinted genes (Bonthuis et al., 2015) in each hypothalamic cell type. Unsupervised hierarchical clustering revealed 24 distinct groups of imprinted genes co-expressed similarly at the cellular level (Figure S7). We found 4 imprinted genes co-expressed with *Ddc,* including *Th*, the noncanonical PEGs *Gpr1* (*G-protein coupled receptor 1*) and *Sec14l3* (*SEC14-like lipid binding 3*), and the canonical PEG *Dlk1* (*Delta-like kinase 1*) (Figure 7F). Thus, a small subset of imprinted genes are top candidates for mediating parental controls over the monoaminergic systems of the hypothalamus in combination with *Th* and *Ddc*.

Finally, we focused on the adrenal medulla, which contains DA-, NE-, and E-secreting chromaffin cells. Triple co-immunolabeling in *Ddc*^*V5/EGFP*^ allelic reporter mice for DA beta-hydroxylase (DBH), the biosynthetic enzyme for NE, and V5 and EGFP revealed that all DDC+ cells, including biallelic-, paternal-, and maternal-allele-expressing cells, co-express DBH and are therefore capable of NE synthesis (Figure 7G). Next, we performed co-labeling with PNMT, the biosynthetic enzyme for E (Figure 7H). We determined that a subset of DDC+-expressing cells also express PNMT. We found that imprinted cells with dominant paternal allele expression are present in both the PNMT+ and PNMT cell populations, composing about ~20%–25% of each, with biallelic expression occurring in the remaining cells (Figure 7I). Therefore, imprinting impacts *Ddc* expression in discrete subpopulations of NE- and E-synthesizing adrenal cells.

## DISCUSSION

We presented multiple lines of evidence that *Th* and *Ddc* noncanonical imprinting is a functional cellular mechanism with roles in determining adult foraging and brain-adrenal axis functions. We show that the maternal and paternal alleles for both *Th* and *Ddc* have different roles in controlling foraging in sons versus daughters. Maternal alleles affect male foraging, while female foraging is under paternal allele control. We show that each parental allele promotes different outcomes (keystone feature level) by affecting the expression of specific and distinct decisions and action sequences during foraging (module level). From *Ddc,* we found that noncanonical imprinting affects allelic expression in subsets of cells in 14 brain regions and the adrenal medulla, and we defined the major brain regions and cell types involved. The maternal versus paternal *Ddc* alleles were revealed to play distinct roles in affecting neural versus metabolic molecular pathways in the adrenal gland, respectively. At the endocrine level, the maternal allele affects DA, NE, and E output in males, while the paternal allele affects DA output in females, which aligns with the pattern of parental influence on the behavior. Therefore, noncanonical imprinting in the monoaminergic system is an ethologically important mechanism of genetic and cellular control over offspring foraging, decision making, and the brain-adrenal axis.

### Noncanonical imprinting effects and the discovery of cell populations determining male and female foraging and brain-adrenal axis phenotypes

The functional significance of noncanonical genomic imprinting effects has been unclear, and the total numbers of affected genes are debated (Gregg, 2017; Perez et al., 2016). Previous work by us (Bonthuis et al., 2015) and others (Perez et al., 2015) showed that noncanonical imprinting (or parental-allele biases) can have important phenotypic consequences. We now show ethological roles for these mechanisms in foraging. For both *Th* and *Ddc,* which reside on different chromosomes, each parental allele controls different foraging modules that are discrete, reproducible chains of decision and action sequences that in turn predict food intake, exposure, distance traveled, and other keystone features of naturalistic foraging. Our approach and insight that parental alleles control foraging decisions in this way builds on (1) Karl Lashley’s insight that behavior is hierarchically constructed with serially ordered action sequences that are pre-determined, not just adaptive responses to sensory inputs (Lashley, 1951; Rosenbaum et al., 2007) and on (2) Golani and colleagues’ work showing that exploratory behavior is stereotypic and naturally segmented by excursions from the home (Drai et al., 2000; Eilam and Golani, 1989). Building on these ideas with a naturalistic foraging assay and an unsupervised machine-learning approach, we now see how genetic mechanisms affect finite foraging modules. Our findings fit with other fields that have concluded that modularity is a fundamental design principle of living systems that enables evolvability (Lorenz et al., 2011; Melo et al., 2016). With this framework, we can now uncover these modules and better understand how parents determine offspring foraging decisions through imprinted genes.

By creating and analyzing *Ddc* allelic reporter mice here, we now have direct cellular- and protein-level evidence that noncanonical imprinting effects can involve differential expression of the parental alleles in specific subpopulations of cells and biallelic expression in other cells. For *Ddc,* many hypothalamic nuclei are affected, as well as midbrain nuclei (e.g., periaqueductal gray [PAG]), which harbor cells with preferential maternal allele expression. The hypothalamus controls a wide range of homeostatic processes, physiological and motivational states, and innate behaviors (Saper and Lowell, 2014). Recent studies show that these diverse functions are mediated by a vast array of molecularly and functionally distinct cell types (Chen et al., 2017; Kim et al., 2019; Moffitt et al., 2018). We found that *Ddc* imprinting effects are enriched in GABA neurons, which are molecularly and functionally diverse with critical roles in modulating the circuit operations that underlie cognition (Huang and Paul, 2019). Thus, these neurons are well suited for mediating imprinted gene controls on foraging and decision making. In the adrenal medulla, preferential expression of the paternal *Ddc* allele was found in subsets of noradrenergic and adrenergic cells, which affect stress responses, metabolism, and glucose homeostasis (Kvetnansky et al., 2009; Verberne et al., 2016). Intriguingly, our adrenal RNA-seq results revealed that the maternal *Ddc* allele affects neural pathways in the adrenal gland while the paternal allele affects metabolic pathways. The hypothalamic, midbrain, and adrenal cells impacted by *Ddc* imprinting effects presumably play important roles in promoting internal states that determine the expression of specific foraging modules in sons and daughters.

We found different roles for *Th* alleles in foraging compared with *Ddc* alleles. We had expected that *Th* and *Ddc* imprinting effects would be functionally additive but found nonadditive functional interactions. One explanation is that despite both being expressed in catecholaminergic cells, *Th* and *Ddc* imprinting effects impact different cell populations that in turn affect different brain functions and monoamine signaling processes. New *Th* allelic reporter lines will be needed to test this. Moreover, by mapping the cells and brain regions showing *Ddc* and *Th* imprinting effects along with those showing effects for *Gpr1*, *Sec1l3*, and *Dlk1—*which are imprinted genes we found to be co-expressed with *Ddc—*a more comprehensive cellular and parental model of foraging control through the monoamine system will emerge.

Our study shows that *Th* and *Ddc* allelic effects on foraging are sexually dimorphic. Moreover, *Ddc* allelic effects on adrenal molecular pathways and brain-adrenal catecholamine outputs differ for males versus females. In both cases, maternal influence predominated in males compared with the paternal influence in females. The mechanistic basis of these sex differences is, so far, unclear. *Ddc* brain and adrenal cellular imprinting effects are present in both sexes and are grossly similar. Thus, sexually dimorphic imprinting effects are not apparent. We found major sex differences in the molecular profile and catecholamine outputs of the adrenal gland, and sex differences in hypothalamic circuitry and gene expression are well known. We speculate that *Th* and *Ddc* imprinting effects functionally interact with molecular and cellular sex differences caused by sex hormones. Future studies will test this by manipulating sex hormones in reciprocal *Th* and *Ddc* heterozygotes. Influences over the male and female brain-adrenal axis could be mediated by imprinting effects impacting sympathetic-adrenal-medullary and/or hypothalamic-pituitary-adrenal pathway functions (Eiden and Jiang, 2018).

Finally, our current approaches have not ruled out the possibility that some phenotypes are indirectly caused by changes to parental behavior or physiology due to *Th* and/or *Ddc* mutant alleles in the mother or father or that important functional interactions take place between the imprinting effects in the offspring and indirect effects from the parents. Nonetheless, our collective findings show the functional importance of the imprinting effects directly in the offspring. The stage is set for future circuit tracing and conditional deletions of the maternal versus paternal alleles in discrete brain regions and cell types to link cellular allelic effects to specific behavioral and physiological components.

### Toward an imprinted gene model of naturalistic foraging and decision control

A major goal of the study of animal foraging has been to develop mechanistic, evolutionary, and ethological models of mammalian decision making, cognition, and behavioral economy (Kalenscher and Wingerden, 2011; Mobbs et al., 2018). Our study supports genomic imprinting as a mechanism of offspring foraging and decision control. Selective pressures for imprinting are proposed to be related to the biased investment of maternal resources in offspring that occurs in mammals (Haig, 2000). Importantly, the transition from nursing to foraging at weaning strongly influences offspring demands on maternal resources (Lee, 1996), setting the stage for selective pressures linking imprinting and foraging. Additionally, some previous experimental support for an imprinted gene model of foraging and decision control exists. We previously found that the Prader-Willi syndrome imprinted gene, *Magel2,* significantly affects foraging modules (Hörndli et al., 2019), building on studies by others showing that *Magel2* affects anxiety-like responses, feeding, activity, and 5-HT signaling (Kozlov et al., 2007; Mercer et al., 2009). Moreover, *Grb10* impacts impulsive choice and risk-taking behaviors (Dent et al., 2016, 2018, 2020). *Grb10* is a canonical imprinted gene that resides next to *Ddc,* and it is paternally expressed in the mouse and human brain and maternally expressed in the body (Arnaud et al., 2003; Menheniott et al., 2008; Sanz et al., 2008). Thus, the *Ddc-Grb10* imprinted gene cluster may have evolved important parental controls over decision making along with the multiple imprinted genes involved in Prader-Willi syndrome. Top-down computational approaches to ethology will be transformative for this emerging area (Bonthuis et al., 2015; Datta et al., 2019). Our current study focused on foraging-module expression frequencies, and future work should also test effects on module expression timing and sequential order. Decision changes over time have been modeled in discounted utility theory, rate maximization, the law of effect, and others (Kalenscher and Wingerden, 2011; Mobbs et al., 2018), and our decomposition of foraging into modules offers the possibility of further understanding genetic and cellular controls on naturalistic decision phenotypes.

### Limitations of the study

Our naturalistic foraging assay captures some important features of the wild context, but owing to the limitations of the lab environment, it is difficult to know how the observed phenotypic effects might manifest in the wild. This limits our ability to draw firm conclusions about the ethological roles of *Th* and *Ddc* imprinting effects. Our approaches to testing parent-of-origin allelic effects on offspring phenotypes are not conditional, which limits our ability to know the developmental stage at which the mutant alleles have their effects and whether some phenotypes are impacted by the mutant allele residing in the parents.

## STAR★METHODS

### RESOURCE AVAILABILITY

#### Lead contact

Further information and requests for resources and reagents should be directed to and will be fulfilled by the lead contact, Christopher Gregg (chris.gregg@neuro.utah.edu).

#### Materials availability

- Plasmids generated in this study are available from the Gregg lab.
- Mouse lines generated in this study are available from the Gregg lab with a Material Transfer Agreement due to University of Utah policy.

#### Data and code availability

- RNA-seq data have been deposited at GEO and are publicly available as of the date of publication. Accession numbers are listed in the key resources table. Microscopy data reported in this paper will be shared by the lead contact upon request.
- All original code is available from the lead contact upon request.
- Any additional information required to reanalyze the data reported in this paper is available from the lead contact upon request.

### EXPERIMENTAL MODEL AND SUBJECT DETAILS

#### Mice

##### Housing and husbandry

All experiments were conducted in compliance with protocols approved by the University of Utah institutional animal care and use committee (IACUC). *Ddc* Allele-Tag mice (see below) were bred and housed on ventilated racks at the University of Utah Comparative Medicine Center on a 12hr light cycle, 6am on and 6pm off; *Th* and *Ddc Het* (see below) mice were bred and housed on static racks in the Biopolymers Building near the lab’s behavioral testing room on a 12hr reversed light, 11pm on and 11am off. All mice were given water and food (Harlan-Teklad 2920X soy protein-free) *ad libitum*, with the exception of a single overnight fast for mice tested for foraging behavior (see method details, behavior, foraging). Adult breeders (6 weeks to 1year of age) were paired continuously, and pups were weaned at postnatal day 21 (P21) and cohoused with up to five same-sex littermates or similar aged same-sex mice of the same line; mice were never singly housed. As needed, ear punches were taken at P7 or P17-P21 for both genotyping biopsy samples and mouse identification purposes. Before dissections of brain, adrenal, and embryonic heart tissue, mice were put to sleep with isoflurane gas anesthesia and decapitated.

##### Ddc Allele-Tag reporter mice

Ddc-6His-P2A-eGFP-3xNLS (*Ddc*^*eGFP*^ line) and Ddc-V5-P2A-mRuby2–3xNLS (*Ddc*^*V5*^) line constructs were designed and assembled by the Gregg lab (see Allele-Tag construction in method details), and the University of Nebraska Medical Center Mouse Genome Engineering Core Facility (Omaha, NE) used these constructs to perform CRISPR mediated homology directed repair for targeted insertion of the reporters immediately before the stop codon of the gene dopa decarboxylase (GRCm38/mm10; chr11:11815230) into C57BL/6J mice (see Easi-CRISPR targeted mutagenesis in method details). Targeted insertion into the genome of F0 founder mice was confirmed by PCR amplifications using primers that flanked the 5′ and-3′ genome integration sites, and sanger sequencing confirmed that the reporter was in-frame with the *Ddc* coding sequence, contained no indels, and no non-synonymous amino acid substitutions.

F0 founder mice were shipped to the University of Utah where they were backcrossed into the C57Bl/6J background strain for five generations to reduce propagation of any potential unknown off-target CRISPR mutations before producing homozygous reporter lines. We found that the mRuby reporter was not detectable and directly labeled for the V5 protein tag to detect expression in the line referred to as *Ddc*^*V5*^. Reciprocal crosses of *Ddc*^*eGFP*^ dams X *Ddc*^*V5*^ sires and *Ddc*^*V5*^ dams X *Ddc*^*eGFP*^ sires produced *Ddc*^*eGFP/V5*^ and *Ddc*^*V5/eGFP*^ offspring, respectively, for microscopy studies. All brains and adrenal glands from reporter mice were collected from adult females (Atlas, P65; AVPV P79–197), and expression of the reporters was restricted to brain regions with known monoaminergic cell populations. Embryonic heart was collected from both sexes between E16–18.

##### Th *and* Ddc *heterozygote mutants*

Germline heterozygous *Ddc* mutant mice were made by crossing CMV-cre (Jax, Stock No: 006054) X *Aadc*^*flox7*^ lines (Zhang et al., 2011). *Aadc*^*flox7*^ mice have loxP recombination sites flanking exon-7 of the *Ddc* gene; this line was rederived at the University of Utah Transgenic Gene-Targeting Mouse Facility by *in vitro* fertilization (IVF) from cryopreserved sperm donated by the lab of Raymond C. Harris. (Vanderbilt University School of Medicine). Exon-7 CRE-recombinant excision was confirmed by PCR genotyping and Sanger Sequencing, and the resulting heterozygous *Ddc*^*Δ7*^ line was backcrossed for 10+ generations into the C57Bl/6J background strain with the CMV-cre transgene removed. The germline *Th* knockout mice were a gift from Dr. Richard Palmiter (University of Washington). A battery of behavioral tests (see behavior section of method details below) of both male and female mice of all genotype groups began between 8–10 weeks of age (P54–75) and lasted for five weeks; one task per week in the same order.

##### Genotyping

Ear punches taken at P7-P21 were lysed in 75μL of 25 mM NaOH +0.25 mM EDTA with a 1 hour incubation in a thermalcycler at 98°C. Lysates were then pH neutralized with an equal volume of 40 mM Tris.HCl, pH5.5. Two μL of lysates were then added to make 20μL PCR reactions with DreamTaq Green Master Mix (ThermoFisher, Cat# K1081) and 0.5μM primers.

### METHOD DETAILS

#### Ddc Allele-Tag reporter mice

##### Recombinant DNA construction

Dopa decarboxycalse (*Ddc*) allele-tag plasmid constructs for knockin to the C57Bl/6J genome were made using the Gibson Assembly method to join *Ddc* homology arms and reporter constructs into the pCRII-TOPO vector. Two custom reporter constructs were designed and synthesized as 1000 ng of ~1kb gBlocks from Integrated DNA Technologies: Ddc40HA-6His-P2A-eGFP-3xNLS and Ddc40HA-V5-P2A-mRuby2–3xNLS (see Table S2C). Ddc40HA-6His-P2A-eGFP-3xNLS consists of the last 40 bp of DDC coding sequence c-terminally conjugated with a 6His epitope tag, P2A self-cleaving peptide sequence, eGFP conjugated with three c-terminal copies of a nuclear localization sequence (3xNLS), stop codon, 37 bp of mutated *Ddc* 3′-UTR (mUTR) to prevent homology repair between the stop codon and a CRISPR cut site 34 bp downstream (preserving the 3′-splice junction of exon 14), followed by 40 bp of un-mutated *Ddc* intron 14 sequence. Ddc40HA-V5-P2A-mRuby2–3xNLS was similarly designed except the *Ddc* c-terminal coding sequence is conjugated with a V5 epitope tag, and the fluorescent reporter is mRuby2–3xNLS.

1000 ng gBlock constructs were diluted in TE to 10 ng/ul. *Ddc* homology arms with approximately 1kb sequence to the left (LHA) and to the right (RHA) of the *Ddc* stop codon were PCR amplified using genomic DNA template isolated from C57Bl/6J by Phenol/Chloroform extraction and ethanol precipitation methods. The 3′-end of the LHA product (Primers: LHA F1, LHA, R1; see Table S2C) ends 1 bp upstream of the stop codon end extends ~1kb in the 5′-direction. The 5′-end of the RHA product (Primers: RHA F1, RHA R1 5’; see Table S2C) begins 40 bp downstream of the stop codon and extends ~1kb in the 3′-direction. For assembly, the entire linear pCRII-TOPO vector (Invitrogen, Cat# K460001) was PCR modified using primers that anneal to the 3′-ends of the open multiple-cloning site, and contain a 40 bp 5′-overhang with either homology to 3′-end of the RHA (PCRIITopo_DdcRHA_F1) or to the 5′-end of the LHA (PCRIITopo_DdcLHA_R1). LHA, RHA, and modified plasmid were PCR amplified using Phusion HF polymerase (New England Biolabs, Cat# M0531S) and purified using EZNA Cycle Pure columns (Omega BiotTek, D6492–01) according to manufacturers’ protocols. In separate 20μL assembly reactions, 75 ng (~0.12 pmol) of Ddc40HA-6His-P2A-eGFP-3xNLS or Ddc40HA-V5-P2A-mRuby2–3xNLS allelic reporter constructs were stitched together with 75 ng (~0.12 pmol) of each homology arm (LHA and RHA) into 100 ng (~0.04 pmol) of the overhang modified pCRII-TOPO vector in Gibson Assembly Master Mix (New England Biolabs, Cat# E2611) for 1hr at 50°C. The Gibson assemblies were then diluted 1:4 in nuclease free water and transformed into One Shot TOP10 (Invitrogen, Cat# C404003) competent *E. coli* cells, plated onto ampicillin selective LB agar plates (supplemented with IPTG and XGAL for blue-white selection), and transformed colonies were picked and grown in 3 mL LB cultures to purify plasmid DNA with EZNA Plasmid Mini Kit (Omega BioTek, D6942–02). Construction of assembled plasmids (pCRII-TOPO-Ddc-6His-eGFP-3xNLS, and pCRII-TOPO-Ddc-V5-mRuby2–3xNLS) was confirmed by sanger sequencing.

##### Easi-CRISPR targeted mutagenesis

*Ddc* Allele-Tag mice were made using the *Easi-CRISPR* methodology at the University of Nebraska, Transgenic Core Facility (Quadros et al., 2017). Briefly, allele-Tag plasmid constructs (pCRII-TOPO-Ddc-6His-eGFP-3xNLS-mUTR, and pCRII-TOPO-Ddc-V5-mRuby2–3xNLS-mUTR) were used as template for PCR amplification using GoTaq Long PCR Master Mix (Promega, Cat# M4021) and ultramer synthetic oligonucleotides (DDC-GFP-IVTRT F and DDC-IVTRT R, and DDC-mRuby-IVTRT F and DDC-IVTRT R; see Table S2C) as primers to make cassettes for use in single-stranded DNA (ssDNA) synthesis by *in vitro* transcription and reverse transcription (IVTRT). PCR products were purified using Wizard SV Gel PCR Clean up System kit (Promega, Cat# A9282). From 5′ to 3′, the cassettes contained 12 buffer nucleotides (atatcggatccc), a T7 transcriptional promoter (TAATACGACTCACTATAG), 78 bp of *Ddc* LHA upstream of the stop codon, the Allele-Tag reporters (either 6His-P2A-eGFP-3xNLS, and V5-P2A-mRuby2–3xNLS), *Ddc* stop codon, 12 bp of mutated 3′UTR to eliminate CRISPR-CaS8 protospacer sequence to prevent re-cutting after homology directed repair, followed by 58 bp of RHA sequence starting 13 nucleotides downstream of the end of the stop codon. RNA was synthesized by *in vitro* transcription using 1.5–2.5 ng of DNA cassettes and mMESSAGE mMACHINE T7 Ultra Kit (Ambion, Cat# AM1354) according to manufacturer’s instructions by incubating overnight at 37°C, followed by adding 1μL of TURBO DNAse (Invitrogen, Cat# AM2238) at 37°C for 15 min, and purified with MEGAclear Transcription Clean-Up Kit (Ambion, Cat# AM1908) according to manufacturer’s instructions. Then cDNA was synthesized using 3–5 μg of RNA and M-MuLV Reverse Transcriptase (New England Biolabs, Cat# M0253S) in 30μL reaction according to manufacturer’s protocol with 90 min 42°C incubation and 5min. 80°C inactivation. RNA was removed by adding 3μL of RNAseH (New England Biolabs, Cat# M0253S) and incubating at 37°C for 30min. Resulting, ssDNAs were gel purified by running on 1% low melting SeaKem Le Agarose (Lonza, Cat# 50002) electrophoresis at 135V for 30min., staining with EtBr and excising ssDNA bands, and extracting from gel slices with Wizard SV columns using 25μL of prewarmed injection buffer for the first and second elution. Injection mix was assembled by mixing Ddc crRNA (see key resources table) and tracrRNA (Integrated DNA Technologies, CAT# 1072534) at 1:2 ratio and annealing in a thermalcycler. 1μL of annealed gRNA (crRNA:tracrRNA) and 0.6μL of CaS8 protein (3.3ug/μL Final Concentration) was mixed into injection buffer and incubate at room temp. for 20–30 min to form ribonucleoprotein (RNP) complexes. Final volume of injection mix is 50μL with final concentrations of 20 ng/μL CaS8 protein (Integrated DNA Technologies, Cat# 1072534) and 10 ng/μL gRNA. Added ssDNA to 10 ng/μL in injection mix and filter through Millipore Centrifugal Filter units (Cat# UFC30VV25) by centrifugation at 13.5rpm for 5min. at room temp. The RNPs and ssDNA were then microinjected into single-cell embryos to cut the genomic DNA approximately 17 bp downstream of the stop codon, and the ssDNA could be used as template for homology directed repair (HDR) and insertion into the genome at the *Ddc* locus. Injected embryos were then grown *in vitro* and implanted into pseudo pregnant females. F0 pups were screened for targeted insertion of the reporter construct by PCR and sanger sequencing across the 5′ and −3′ ends of the RHA and LHA to ensure integration into the genome.

#### Histology

##### Tissue preparation

Under deep isoflurane anesthesia, adult reporter mice were transcardially perfused with PBS, to clear blood, followed by ~25–50 mL of 4% paraformaldehyde to fix tissues. After perfusion, brains were dissected from the skull and adrenals from the abdomen, post-fixed in 5 mL of 4% paraformaldehyde for ~48 hrs at 4°C, cryoprotected by immersing serially in 15% and then 30% sucrose at 4°C until the tissues sank, embedded and frozen into Optimal Cutting Temperature Compound (OCT) in cryomolds using a methanol dry-ice bath, and stored at −80°C until sectioning. Coronal brain sections from regions of interest were then cut on a cryostat at 20 μm thickness to directly mount onto Superfrost Plus slides (Fisher, Cat #12–550-15), air-dried, and stored in a slide box at −80°C until Nissl stained or immunolabelled. Brain and adrenal tissue labeled as floating sections were processed as above, except they were cut at 30 μm thickness, collected into vials of antifreeze solution (300 g sucrose, 10 g polyvinylpyrrolidone, 300 mL ethylene glycol, and 0.1 M phosphate buffer to 1L) and stored at −20°C until immunolabeling.

For embryonic heart tissue, pregnant mothers were euthanized under isoflurane anesthesia before dissecting out the uteri containing embryos into ice-cold PBS in a Petri dish. Embryos were then dissected from the uterus and extraembryonic tissue and transferred to a clean dish with fresh PBS. Embryonic hearts were micro-dissected under a stereo microscope, immersed in 4% paraformaldehyde for ~48hrs at 4°C, exchanged with 15% and 30% sucrose, frozen in OCT molds made from aluminum foil, and stored at −80°C. 10 μm embryonic heart sections were cut on a cryostat, mounted onto Superfrost Plus slides, and stored in a slide box at −80°C until staining.

##### Nissl stain

For the brain Atlas of *Ddc* maternal dominant expressing cells, adjacent parallel sections were Nissl stained to identify neuroanatomical landmarks according to the Allen Mouse Brain Reference Atlas. Slides with 20 μm sections were immediately submerged in 1:1 ethanol/chloroform overnight, then rehydrated through 100%, 95%, 70%, 50% ethanol to distilled water. Sections were then stained for 10 min in prewarmed 0.1% cresyl violet made fresh with 0.3%v/v glacial acetic acid in a 45°C oven. Slides were then quickly rinsed in distilled water and differentiated for 2–30 min in 95% ethanol while checking microscopically for best result. Sections were then dehydrated in 100% ethanol 2 × 5min., cleared in xylenes 2 × 5min, and cover-slipped with EcoMount (Biocare Medical, Cat# EM897L) mounting medium.

##### Immunolabelling

Slides with 20 μm brain or adrenal sections (Figures 2I, S2, and S3) were removed from −80°C storage, air-dried and hydrophobic barrier pen was drawn around sections. By Immersing slides in Copland jars on an orbital shaker with gentle-agitation, sections were hydrated in PBS, permeabilized for 10min. in PBS +0.2% triton-X 100 and washed 3 X 5 min. with PBS + 0.025% triton-X 100. In a humidified chamber sections were covered and blocked with 10% normal donkey serum (Lampire Biological Products, Cat# 7332100) and 1% BSA in PBS for 2hr. at room temp., and then incubated overnight at 4°C with goat polyclonal anti-V5 primary antibody (Abcam, Cat# ab9137) diluted 1:1000 in primary buffer (1% BSA in PBS). The next day, sections were washed 3 X 5 min with PBS +0.025% Triton-X 100 in Copeland jars at room temp. with gentle agitation. Then, sections were covered with donkey anti-goat-568 or 647 (Invitrogen, Cat #A32816, Cat #A-11057) diluted 1:250 in PBS (no triton) and incubated for 2 hr. at room temp. protected from light in a humidified chamber. Sections were then washed 3 X 5 minutes with PBS, and cover-slipped with Vectashield anti-fade mounting media with DAPI (Vector Labs, Cat# H-1200). Embryonic heart sections (Figures 2B–2G) were immunolabelled similarly except they were cut to 10 μm thickness, blocked for 30 min with 10% donkey serum (no BSA), primary buffer contained 10% donkey serum (no BSA), secondary was diluted 1:200, and sections were cover-slipped with ProlongGlass + DAPI (ThermoFisher, P36981).

Floating sections were transferred from vials to wells of a 12-well polystyrene microtiter plate for immunolabelling; plates were placed on an orbital shaker to gently agitate sections for all washes and incubations. To remove antifreeze, sections were fist washed with 6 × 5 min. PBS exchanges. Then, sections were blocked with 10% normal donkey serum + 0.3% Triton-X 100 for 1 hour at room temperature, and incubated with primary antibodies (chicken anti-GFP 1:2000, Abcam Cat# ab13970; goat anti-V5 1:1000, Abcam Cat# ab9137; rabbit anti-GABA 1:500, Sigma Cat# A2052; Sheep anti-PNMT 1:200, R&D Systems #AF 7854; Rabbit anti-DBH 1:100, Immunostar #22806) overnight at 4°C. The next day, sections were washed 3 X 20 min with 0.1% Triton-X 100 in PBS exchanges. While protecting from light, sections were then incubated with secondary antibodies (donkey anti-chicken-488 1:200, Jackson Immuno Cat# 703–545-155; donkey anti-rabbit-568 1:200, Invitrogen Cat# A10042; donkey anti-goat-647 1:200, Invitrogen Cat# A-21447) in 1% normal donkey serum +0.1% Triton-X 100 in PBS for 2 hrs. at room temp., washed for 3 X 20min. with 0.1% Triton-X 100 in PBS, and finally washed in 2 PBS exchanges. Using a paintbrush and 0.5X PBS, sections were float-mounted onto positively charged slides, and cover-slipped with ProlongGlass + DAPI mounting media.

##### DDC brain atlas

The entire fixed brains of *Ddc*^*GFP/V5*^ and *Ddc*^*V5/GFP*^ adult female mice were sectioned at 20 μm thickness and mounted onto positive charged slides in five parallel series containing representative coronal sections along the entire rostral caudal axis. Every section from one series for both crosses was fluorescently immunolabled for V5 as described above to detect cells expressing the *Ddc*^*V5*^ allele, while expression of the *Ddc*^*GFP*^ allele was visualized by native GFP fluorescence concentrated in the nucleus. A second series with parallel sections to the immunolabelled series was Nissl stained. Epifluorescence images of DDC + cell clusters were captured on a Zeiss AxioImager 2 as optical slices using ApoTome2 structured illumination, a 20X objective, fluorescence filters (DAPI, GFP/488, TexasRed/568, 647) and Zen2 software. Nissl stained sections were captured with brightfield illumination, a 5X objective, and multiple images capturing entire coronal sections were stitched together using a motorized stage and Zen2 tiling. The fluorescence images captured in the camera’s 20X field of view were compared to an adjacent, parallel Nissl stained section and the Allen Brain Reference Atlas to identify and label the anatomical location(s) of the DDC + cells (Figures 3A and 3B). Images were then assigned a random number and a researcher blind to cross and anatomical location manually scored whether the images contained GFP dominant, GFP biased, biallelic, V5 biased, and/or V5 dominant DDC expressing cells. Sections from AVPV of five *Ddc*^*GFP/V5*^ and five *Ddc*^*V5/GFP*^ adult female were prepared as for the Atlas and a researcher blind to genotype and cross counted the total number of all GFP and V5 biased and dominant allele expressing, and biallelic cells in every image of the region.

#### ELISA

##### Urine collection and analysis

Spontaneous urine samples were collected from *Ddc*^−/+^, ^+/−^ *and* ^+/+^ adult mice (2–6 months old) 2 hours after lights off on consecutive days until 3 repeated samples per mouse were collected. Mice were given 60 seconds to urinate into a Petri dish per day for each attempt. Each individual urine sample was split into 2 tubes. One tube contained >3 ul for creatinine analysis and the second contained >7ul for catecholamine ELISA tests. Equal parts 0.01N HCL were added to each tube and the samples were stored in −80C until analysis. For analysis, the samples were pooled with identical volumes from each. ELISA analysis for Epinephrine, norepinephrine and dopamine were performed using the Eagle Biosciences kit (#KA1880). Creatinine analysis was performed using the Arbor Assays Kit (#K002-H1). Sample analysis was performed on a plate reader. ELISA readings were normalized to creatinine levels and analyses were performed in PRISM 9 (Graphpad) using the provided ELISA workflow.

##### Adrenal tissue collection and RNA-Seq

Whole adrenal glands were collected from *Ddc*^−/+^, ^+/−^ *and* ^+/+^ adult male and female mice (2–3 months old). RNA was extracted using the QIAGEN RNeasy Kit with DNAse treatment. Whole transcriptome sequencing was performed using the RiboZero TruSeq Stranded Total RNA Library Prep Kit (Illumina). Samples were deep sequenced on an Illumina NovaSeq 6,000 system to a depth of 25 million paired-end reads per sample (n = 4). Analyses were done using DESeq2 and edgeR Bioconductor packages in R.

#### Behavior

Mice were tested once per week between 11am-6pm (dark phase) for five weeks in the following order of behavior tasks: foraging, week 1; open field, week 2; elevated zero maze, week 3; light-dark box, week 4; and social preference, week 5. Bedding was left unchanged for 5–8 days prior to each test. Mice were transferred in their home cages and habituated to the testing room with the door closed and ambient room lights off for at least 30min. prior to any test. All test arenas were placed on an elevated stage encircled by black curtains with four cameras, and infrared and white light (when needed) fixtures mounted on a pole high above (~6ft) the stage. One, two, or four mice were tested at a time depending on test (see below). Mice movements were tracked with Ethovision XT 14 software (Noldus) under infrared illumination to collect behavioral data. Each arena was recorded by an individual camera that was centered and zoomed on the arena to fill the entire image space, focused on the subject, and aperture optimally opened to both enhance contrast of the black subject with background and to eliminate oversaturated glare from overhead light reflections. Subject identification numbers and independent variables of sex, age, and genotype were recorded into Ethovision with each test mouse. After testing, mice were housed into a new clean cage with a handful of soiled bedding from their previous home-cage. Between subjects, and before first trial, behavioral apparatuses were wiped-down with 70% ethanol to clean and remove odors, and males were always tested before females on any given day of testing. After Ethovision captures videos and tracks mouse movements, an investigator used the *Tracking Editor* to correct missed samples and tracking errors. The current study focuses on foraging behavior, but significant *Th* and *Ddc* imprinting effects on behavior were independently confirmed in the other exploratory and social behavior tests performed (Bonthuis and Gregg, unpublished data).

#### Foraging

Foraging behavior testing was performed as detailed previously (Hörndli et al., 2019). In brief, in preparation for the foraging assay, mice were first habituated with sand (Jurassic play sand, Jurassic Sand) and seeds (Whole millet, Living Whole Foods) for two days in their home cage. On day one, seeds are spread on top of sand in the bottom of a Petri dish and the dish is placed on the bedding in the home cage for the mice and pups to explore. On day two, seeds are covered with sand in the bottom of the Petri dish in the home cage for the mice and pups to dig in and explore. To motivate animals to feed, mice were food deprived prior to testing to achieve 8%–10% weight loss at the time of testing. We selected this weight loss target after several pilot studies with the goal of achieving some consistency in the motivational states of the animals at different ages and not compromising health or activity. To achieve the intended weight loss and motivational state, adult mice were food deprived for 24 h. Water is available *ad libitum* at all times except when mice are in testing cage (2 × 1 hour).

Mice are housed in a room with an 11:00 – 23:00 dark cycle, so that testing is performed during the dark cycle. For testing, mice are moved into the behavior room prior to the start of testing for at least 1 hour for habituation to the new room. All testing is performed in the dark and video recording is done using infrared illumination and all manual procedures are done in the dark using red light. The mouse to be tested, and their home cage soiled bedding, are moved to the testing-cage and allowed to habituate. At the start of testing, the testing-cage is attached to the arena via the tunnel, the mouse now has access to the arena and video recording starts for the Exploration phase. Mouse behavior is recorded continuously during the 30 min Exploration phase trial under infrared lights. Noldus Ethovision software v14 were used for video tracking. After completing the Exploration phase, the mouse is returned to the testing cage with water but no food until the Foraging phase four hours later. For the Foraging phase, the testing cage is then gently attached to the tunnel and access to the arena is possible and the Foraging trial begins. Video recording of the Foraging phase is performed for 30 minutes. After testing, mice are placed in a new cage with food and are returned to the mouse colony room. Between each Exploration and Foraging phase trial, the entire arena, including walls, platform, tunnel, and steel pots, are wiped clean with 70% ethanol.

##### Preparation of sand and seed pots for the foraging assay

Three stainless steel pots (Resco, diameter 5.5cm, depth 4cm) were filled with 95 g of sand. For the Exploration phase, 1 pot is filled with 80 g of sand covered with 2.5 g of seeds. On top of seeds, a layer of 12 g of sand is added to cover seeds. This sand is then covered with 0.5 g of seeds. This pot is placed in position 2 in the arena. For the Foraging phase, 1 pot is filled with 80 g of sand, 3 g of seeds on top of sand and additional 12 g of sand to cover all seeds. This pot is placed in position 4 in the arena. All pots are weighed before and after the trial to measure the sand displaced from each pot. Remaining seeds and hulls left in the pot and on the platform are measured after each Exploration or Foraging trial to determine the amount of seeds consumed by the mouse during the trial. Used sand is collected after every trial and set aside. At the completion of all testing, the used sand is autoclaved before reuse in future trials.

##### Foraging arena construction

The foraging arena, tunnel, and testing cage were custom built with acrylic plastic (Delvies Plastics, Salt Lake City, UT, USA). The 14 cm long tunnel enters the platform from underneath through one of the five 5.5 cm diameter holes in the arena platform. The platform is 8 cm above stage level and is made from 0.5 cm thick white Plexiglas. The arena is made from a transparent Plexiglas tube and the 0.4 cm thick walls raise 42 cm above the platform. The arena has a diameter of 35 cm. The walls of the arena were roughened with sand paper to limit glaring and recording artifacts.

##### Arena tracking zones

The arena is organized into zones that are used to breakdown the behavior and foraging strategies used by each animal in the assay. The arena is divided into five sectors and the boundary of each sector is the mid point between two pots. The arena is further divided into three concentric circles, including the middle center zone, the intermediate zone and the outer wall zone. The outer radius of the *Intermediate* zone intersects with the center of the pots and tunnel entry. The radius of the *Center* zone is half the radius of the *Intermediate* zone. A zone is also created around each pot in the arena. *Pot* zones have a radius of 1.7x the radius of the pot itself. Finally, to learn about the behavior of the animal related to entries to and exits from the arena, we define zones around the tunnel entry. The *Tunnel Entry* zone aligns with the entry hole of the actual tunnel. The *In Tunnel* zone is covering the most peripheral area of the *Tunnel Entry* and tracks the mouse just before leaving the arena completely. Whenever the mouse is in the cage, the tracking system is recording the mouse as being in the *In Tunnel* zone. The *Tunnel Zone* area has the same radius as the *Pot* zones.

##### Automated tracking

At the start of the trial, the tracking begins with a 10 s delay to allow time for the connection of the testing-cage to the arena. The mouse is first tracked when it appears in the *In Tunnel zone* and position and movement is continuously recorded after this time point until the end of the 30 min Exploration or Foraging Phase. The XY position of the center of the mouse is video tracked with at a rate of 30 frames per second. For the data analysis, tracking data from 0–25 minutes are used. Time spent in each zone, latency to visit a zone and number of visits to each zone, as well as the distance traveled, are calculated using the Ethovision software. The data are exported as results for the total 25-minute trial duration, as well as in 5 minute time bins. Sand displacement and food consummation measures are collected and calculated manually.

##### Behavioral measures

All measures captured during Exploration or Foraging phases of the assay are presented in (Hörndli et al., 2019). All ‘time spent in zone’, sand displaced, food consumed, and zone visit measures were normalized to the total time spent in the arena (TTA) by dividing each value by TTA (x/TTA). For time bin values, the TTA for the corresponding time bin is used for normalization (i.e., x-1/TTA-1). All latencies to visit pots were normalized to the latency to enter the platform (LEP) by subtracting the LEP (x-LEP). Latency to the center zone after arena entry (LCAE) is already normalized to LEP and does not need any further normalization. All percentage measures do not need any normalization. Time on the platform itself and time in the tunnel (cage) are not included in the normalized dataset because they are closely related and redundant to TTA.

##### Locomotor measures

The raw data files generated by Noldus Ethovision listing all XY coordinated and all zones as well as distance traveled and velocity for each frame were used to extract data describing excursions and locomotor patterns using custom code, including the duration and number of bouts at different velocities. An excursion is defined as beginning when the mouse leaves the tunnel (In Tunnel zone) and ending when the mouse returns to the tunnel (one round trip). Continuous velocity values in the data were categorized into three velocity classes, *slow*: velocity ≤5 equals; *medium*: velocity >5 ≤ 15; *fast:* velocity >15. The length of the bout is calculated using the number of frames in the sequence and all sequences of the same velocity class longer than 3 frames are counted as a single velocity bout.

### QUANTIFICATION AND STATISTICAL ANALYSIS

#### Genomics

##### Imprinting ~ expression correlation network analysis

Published RNASeq maternal and paternal allele expression data and gene expression data for the arcuate nucleus region of the adult female hypothalamus for F1cb (n = 9) and F1bc (n = 9) mice was obtained (Bonthuis et al., 2015). For each biological replicate, the difference in the expression of the maternal and paternal allele was computed for each gene (maternal – paternal). The correlation of the maternal and paternal allele expression difference to the expression of published GABA and glutamate neuron hypothalamic cell-type marker genes was computed (Chen et al., 2017). We then tallied the number of positively (r > 0) and negatively (r < 0) correlated marker genes for each cell-type category and a Chi-square test of the dependence of the allelic expression difference on the cell type category was computed. The mosaic plots were computed using the vcd package in R, which revealed the pearson residuals and associations between cell-types and parental allele expression.

##### Single cell RNASeq data analysis

Published adult mouse hypothalamus single cell RNASeq data generated by (Chen et al., 2017b) were downloaded from GEO (gene expression omnibus). We extracted the gene expression data for each cell assigned to each cell-type, according to definitions in the published study. The mean expression level for each gene was computed across all cells for a given type. We then extracted the data for all cell types and imprinted genes, based on imprinted genes identified in our previous study (Bonthuis et al., 2015). Unsupervised hierarchical clustering of imprinted gene cellular expression profiles was performed in R using Euclidean distance and the Ward.D2 method.

#### Brain regions with DDC imprinted cell subpopulations

##### Atlas

After determining whether each image exclusively contained subpopulations of cells with dominant GFP or dominant V5 allelic expression, or not, they were decoded for cross and brain region and organized into 52 groups according to brain region(s) for statistical analysis (n > 7 per region). Fisher’s Exact tests determined for each region whether there was a significant difference in the number of images from *Ddc*^*GFP/V5*^ compared to *Ddc*^*V5/GFP*^ containing GFP dominant cell subpopulations; the same was done for subpopulations of V5 dominant cells. Each region was then plotted with X- and Y-coordinates of Figure 3C, with the X axis representing the −log(p) value for a cross difference in GFP dominant subpopulations and on the Y axis representing the −log(p) for a cross difference in V5 dominant subpopulations. On both axes the −log(p) values were plotted in the positive direction when allele dominant expression comes from the maternal allele (i.e. from GFP of the *Ddc*^*GFP/V5*^ cross, and from V5 of the *Ddc*^*V5/GFP*^ cross) and in the negative direction when allele dominant expression comes from the paternal allele (i.e. from GFP of the *Ddc*^*V5/GFP*^ cross, and from V5 of the *Ddc*^*GFP/V5*^ cross). Therefore, regions in the upper right coordinate of the plot represent regions that show dominant maternal allele expression from both crosses.

##### AVPV

Counts of the number GFP dominant (GD), GFP biased (GB), biallelic (BA), V5 biased (RB), and V5 dominant (RD) cells from all images of the AVPV were calculated as percent of the total number of all DDC + cells for each individual; n = 5 *Ddc*^*GFP/V5*^ and n = 5 *Ddc*^*V5/GFP*^. These data were analyzed with a two-way ANOVA with *Cross* and *Cell-type* main factors in GraphPad Prism 5 software.

#### Foraging behavior

##### Excursion data capture

Our DeepFeats approach for analyzing modularity in foraging was performed as previously described (Hörndli et al., 2019) with some modifications and advances. In our study, mice were tracked with Noldus Ethovision software. Noldus settings were used to define regions of interest in the foraging arena and indicated when the mouse was in each area. To ensure the tracking is equivalent across different mice, a Procrustes transformation of the XY coordinates was performed to put every tracking file in the same coordinate space. The track coordinates were zero’d to the center of the tunnel to the home cage. We then generated custom code in R to parse the raw Noldus tracking files into discrete, round trip home base excursions from the home cage tunnel. Each excursion is assigned a unique ID key that we call the Concise Idiosyncratic Module Alignment Report (CIMAR) string key. It stores the coordinates of the excursion in the data and the CIMAR string includes metadata regarding the mouse number, excursion number, sex, age, genotype, and phase. Next, custom code compares the CIMAR coordinates to the raw Noldus data files and constructs a new dataset that extracts 57 measures from the Noldus output, which we use to initially statistically describe each excursion. The 57 measures are presented in (Hörndli et al., 2019) and are designed to capture a relatively comprehensive array of different behavioral and locomotor parameters, as well as describe interactions with food and non-food containing patches and exposed regions in the environment. These measures consist of shape, frequency, order, and location statistics of an animal’s X and Y movements, numbers of visits and time spent at different features in the arena, including food patches (Pots#2 and 4), non-food containing patches (Pots#1 and 3), the tunnel zone, wall zone, and center zone of the arena and data describing locomotor patterns, including velocity, gait, and distance traveled. The 57 measures for each excursion are scaled (normalized and centered across excursions) because they are in different units.

##### Behavioral measures to resolve modularity in excursions

This section details the methods to define the set of behavioral measures that best resolve candidate modules. A data matrix was constructed in which the rows are excursions performed by the mice, labeled by CIMAR keys, and the columns are the 57 behavioral measures. A correlation matrix was constructed from the data using the Pearson correlation statistic. The measures were then systematically filtered from the data as detailed in the main text based on different correlation thresholds using the “findCorrelation” function in the caret package in R. With this approach, the absolute values of pairwise correlations are considered. If two variables have a high correlation, the function looks at the mean absolute correlation of each variable and removes the variable with the largest mean absolute correlation. We systematically threshold the data in r = 0.5 + value increments as shown in the main text to identify the best set of measures to resolve clusters of excursions. At each threshold, the retained measures are used in an unsupervised clustering analysis to define clusters of excursions. We used the Ward.D2 minimum variance method implemented using the “hclust” function in R to perform the clustering and define compact, spherical clusters. We then statistically define discrete excursion clusters from the results using the Dynamic Tree Cut algorithm (Langfelder et al., 2008). This is a powerful approach because it is adaptive to the shape of the dendrogram compared to typical constant height cutoff methods and offers the following advantages: (1) identification of nested clusters; (2) suitable for automation; and (3) can combine the advantages of hierarchical clustering and partitioning around medoids, giving better detection of outliers. We detect clusters using the “hybrid” method and use the DeepSplit parameter set to 4 and the minimum cluster size set to 20. The total number of clusters detected is quantified at each correlation threshold. Conceptually, more relaxed correlation threshold cutoffs could reduce cluster detection by retaining redundant measures that mask important effects from other measures. On the other hand, thresholds that are too stringent could reduce cluster detection by pruning informative measures. Our objective is to identify the threshold that uncovers the most informative and sensitive set of measures for resolving different clusters of excursions, setting the stage for the discovery of potential modules.

##### Statistical validation of significant clusters of excursions

In our study, Dynamic Tree Cut will deeply cut branches in a dendrogram generating large numbers of small clusters if there are few bona fide relationships in the data. Thus, to test whether bona fide clusters of excursions exist in the data we implemented a random sampling procedure in R in which we randomly sample from the matrix of the retained behavioral measure data to break the relationships between the excursions and the measures. The sampled null data matrix is then subjected to the same clustering and quantification procedure to determine the number of clusters found by Dynamic Tree Cut. A null distribution is created from 10,000 iterations and compared to the observed number of clusters, which is expected to be significantly less than the null due to bona fide biological relationships between the excursions and set of retained measures. A lower tailed p value was computed to test this outcome. In a modification compared our previous study.

##### IGP permutation test

To test whether reproducible modules of behavior exist in the data for the foraging excursions, we use the in-group proportion (IGP) statistical method for testing for reproducible clusters between two datasets (Kapp and Tibshirani, 2007). We built a modified version of this function for parallelized computing to speed the analysis for large numbers of permutations. The excursion data for the mice is separated into a training data and test data partition for reproducibility testing. A balanced partition was generated according to genotype, sex, and phase factors using the “createDataPartition” function in the caret package in R. Unsupervised hierarchical clustering was performed on the Training data partition excursions and clusters were defined using Dynamic Tree Cut. Next, the centroids for each training data cluster were computed as the mean values of the behavioral data for the excursions in the cluster. The training data centroids were then used to compute the IGP statistic for each training data cluster based on the test partition data, thereby evaluating the reproducibility of each cluster.

In a modification compared to our previous study (Hörndli et al., 2019), we created a custom IGP permutation test that is based on a distance calculation, rather than the correlation implementation in the clusterRepro R package. The distance IGP testing framework was written in C++ and speeds the permutation test by many fold and is a more accurate replication of the clustering parameters used in the test data. We used this approach to compute p values for each cluster to determine whether the IGP value is greater than chance. False positives due to multiple testing errors were controlled using the q-value method (Storey, 2002). Modules are thus defined as significantly reproducible training partition excursion clusters (q < 0.1). Each module detected was assigned an ID number and individual excursions in the data were annotated based on the module they match to. This approach facilitated quantifications of module expression frequency by the mice.

##### Statistical modeling of module expression counts

To statistically evaluate the genetic and parental factors that significantly affect module expression frequency, we used generalized linear modeling functions implemented in R. The hypotheses tested are detailed in the text for each analysis and below. Prior to module-by-module statistical testing, we filtered based on overall variance to remove modules with low expression variance across all mice in the study and reduce multiple testing errors, which is a proven two-step method for analyzing high dimensional data (Bourgon et al., 2010). Following subsequent module-by-module statistical testing, p values were corrected for multiple testing errors by the q-value method to control the false discovery rate and pi0 was computed to determine true nulls in the data (Storey and Tibshirani, 2003). In all cases, true nulls included less than half of the modules in the data.

Generalized linear models were fit to maternal or paternal cross data for sons or daughters using the *glm* function in R with the factor of Genotype (levels: wildtype, *Th het*, *Ddc het*, *ThDdc het*). The anova function in R was then used to generate Analysis of Deviances Tables with p values to determine whether genotype main effect explained a significant amount of the variance in the module count data. Generalized linear modeling was performed using a Poisson distribution. We tested the goodness-of-fit of the Poisson for each module with a chi-square test of the residual deviance and degrees of freedom in R. If the goodness-of-fit test indicated the data fit the Poisson model (p > 0.05), we proceeded. All modules passed the goodness-of-fit test.

For statistical tests of cross effects (generalized parental effects), genetic effects (genetic effects independent of cross) and putative imprinting effects (cross × genotype interactions), the *glm* included the terms *Cross* (levels: maternal, paternal), Genotype (levels: wildtype, *Th het*, *Ddc het*, *ThDdc het*) and the interaction effect (*Cross:Genotype*) (*ie. module counts ~ cross + genotype + cross:genotype*, family = ”poisson”). Generalized parental effects are cases with a significant main effect of cross in the absence of a significant cross X genotype interaction. Genetic effects independent of cross have a significant main effect of genotype in the absence of a significant cross X genotype interaction. Putative imprinting effects have a significant cross X genotype interaction effect.

Fisher’s Exact testing for categorical enrichments was performed using the *fisher.test* function in R.

##### Statistical modeling of module transitions

To statistically evaluate transitions between modules, the start time for each expressed module was recorded. We then constructed 1-step transition matrixes that relate the expressed module to the next expressed module to examine sequences of module expression. To determine whether module expression transitions are dependent on the identity of the previously expressed module, we performed a Fisher’s Exact test on the transition count matrix. To test the null hypothesis that female module transitions are not significantly different from male module transitions, we computed the transition matrices separately for male and female data. To compare them, we computed the stationary probability distribution and then the Euclidean distance between the male versus female distribution. Next, we compared the observed distance to a distribution of distances derived from randomly permuted data. In the permutation test, modules are randomly sampled from the male and female data, two transition matrices are generated, the stationary probability distribution is computed and then compared by Euclidean distance. The observed distance is compared to the permuted distribution (>10,000 permutations) to determine the p value.

## Supplementary Material

9

8

7

6

5

4

3

2

1

## Figures and Tables

**KEY RESOURCES TABLE:** 

REAGENT or RESOURCE	SOURCE	IDENTIFIER

Antibodies		

Goat polyclonal anti-V5 tag	Abcam	Cat#ab9137; RRID: AB_307037
Rabbit anti-GABA	Sigma-Aldrich	Cat#A2052; RRID:AB_477652
Chicken polyclonal anti-GFP	Abcam	Cat#ab13970, RRID:AB_300798
Sheep anti-PNMT	R&D Systems	AF 7854
Rabbit anti-DBH	Immunostar	22806

Bacterial and VIRUS strains		

Top10 chemically competent *E. coli*	Invitrogen	Cat#K460001

Chemicals, peptides, and recombinant proteins		

2X Gibson Assembly Master Mix	New England Biolabs	Cat#E2611
mMESSAGE mMACHINE T7 Ultra Kit	Ambion	Cat#AM1354
S.p. Cs9 Nuclease 3NLS	Integrated DNA Tech.	Cat#1072534

Critical commercial assays		

PyroMark Gold Q24 Reagents (5 ×24)	Qiagen	Cat#970802
Catecholamine ELISA kit	Eagle Biosciences	KA1880
Creatinine Kit	Arbor Assays	K002-H1

Deposited data		

Adrenal RNA-Seq data	NIH Short Read Archive	PRJNA800471

Experimental models: Organisms/strains		

Mouse: C57Bl/6J	The Jackson Laboratory	JAX: 000664
Mouse: *ThΔ*	Richard Palmiter	University of Washington
Mouse: CMV-Cre: B6.C-Tg(CMV-cre)1Cgn/J	The Jackson Laboratory	JAX: 006054
Mouse: *Aadc*^*flx*^: B6.129-Ddc^tm1.1Rhrs^	Zhang et al., 2011	MGI:5296328
Mouse: *Ddc*^−/+^, *Ddc*^+/−^	This paper	
Mouse: *Ddc*^*GFP*^: C57Bl/6J-*Ddc*^*em1(6His-P2A-eGFP–3×NLS)*^	This paper	
Mouse: *Ddc*^*V5*^: C57Bl/6J-*Ddc*^*em2(V5-P2A-mRuby2–3×NLS)*^	This paper	

Oligonucleotides		

Primers for genotyping, see Table S2A	This paper	N/A
Primers and Ultramers for Ddc Allele-Tag Constructs, see Table S2C	This paper	N/A
Custom CRISPR-Cas9 crRNA: Ddc crRNA: AAUGAAAGCAGAGCUGCUUC	This paper; Integrated DNA Technologies	
Alt-R CRISPR-Cas9 tracrRNA, 5 nmol	Integrated DNA Tech.	Cat#1072532

Recombinant DNA		

pCRII TOPO	Invitrogen	Cat#K460001
Custom gBlocks gene Fragments;	This paper; Integrated DNA	N/A
Ddc40HA-6His-P2A-eGFP-3×NLS, see Table S2C	Technologies	
Custom gBlocks gene Fragments;	This paper; Integrated DNA	N/A
Ddc40HA-V5-P2A-mRuby2-3×NLS, see Table S2C	Technologies	
Plasmid: pCRII-TOPO-Ddc-6His-eGFP-3×NLS	This paper	N/A
Plasmid: pCRII-TOPO-Ddc-V5-mRuby2-3×NLS)	This paper	N/A

Software and algorithms		

R		
Ethovision XT 14	Noldus	
Prism 5	GraphPad	

Other		

Lme4 R package	CRAN	
DeepFeats custom software	Gregg Lab	

## Highlights

- Maternal versus paternal *Ddc* and *Th* alleles have distinct ethological roles
- Maternal alleles affect sons and paternal alleles affect daughters
- Each parental allele controls specific decision sequences and underlying cells
- *Ddc* parental alleles differentially affect brain-adrenal axis function
