# Supplementary figures and images for: Noncanonical genomic imprinting in the monoamine system determines naturalistic foraging and brain-adrenal axis functions
Source: Cell Rep. Author manuscript; Available in PMC 2022 May 24. (PMC9128000; doi:10.1016/j.celrep.2022.110500)

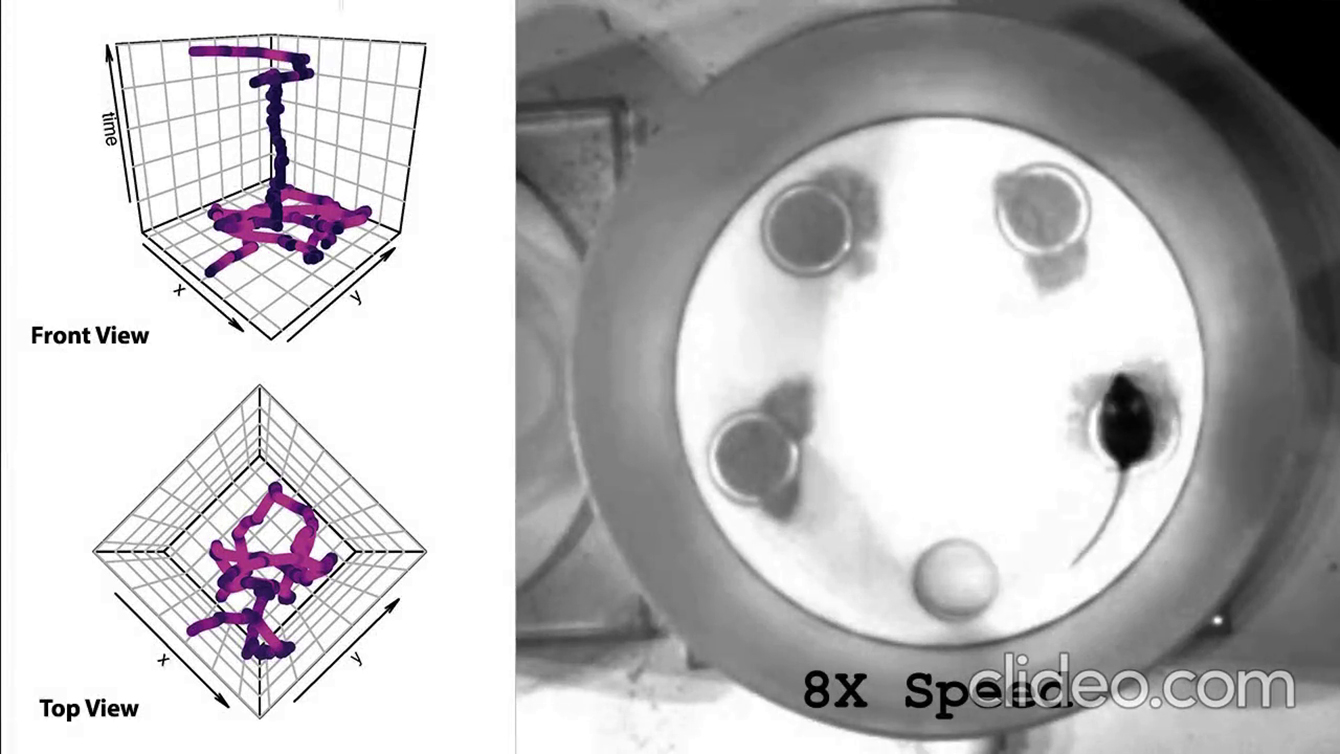

Supplement: 8 [file NIHMS1787429-supplement-8.jpg]

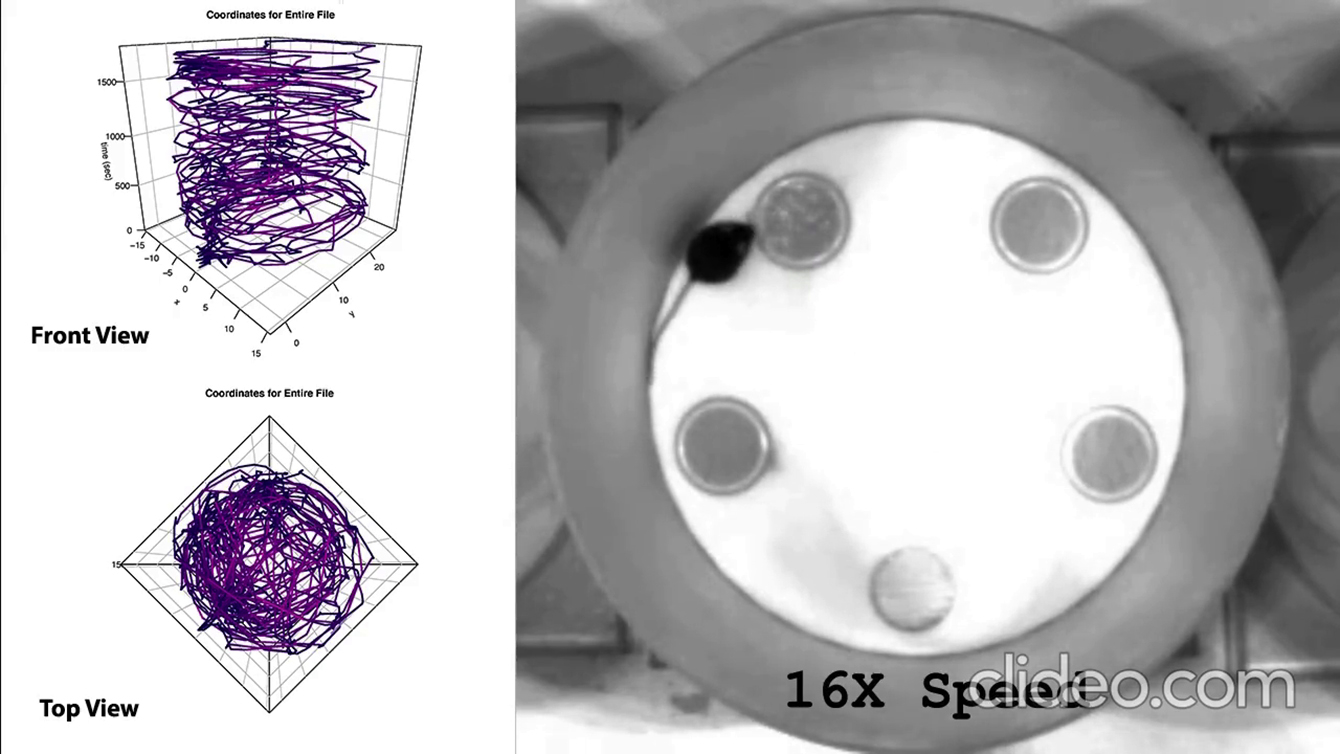

Supplement: 6 [file NIHMS1787429-supplement-6.jpg]
