# Supplementary material for: Noncanonical genomic imprinting in the monoamine system determines naturalistic foraging and brain-adrenal axis functions
Source: Cell Rep. Author manuscript; Available in PMC 2022 May 24. (PMC9128000; doi:10.1016/j.celrep.2022.110500)
Supplement: 1 [file NIHMS1787429-supplement-1.pdf]

Cell Reports, Volume *38*

Supplemental information

Noncanonical genomic imprinting in the monoamine system determines naturalistic foraging and brain-adrenal axis functions

Paul J. Bonthuis, Susan Steinwand, Cornelia N. Stacher Hörndli, Jared Emery, Wei-Chao Huang, Stephanie Kravitz, Elliott Ferris, and Christopher Gregg

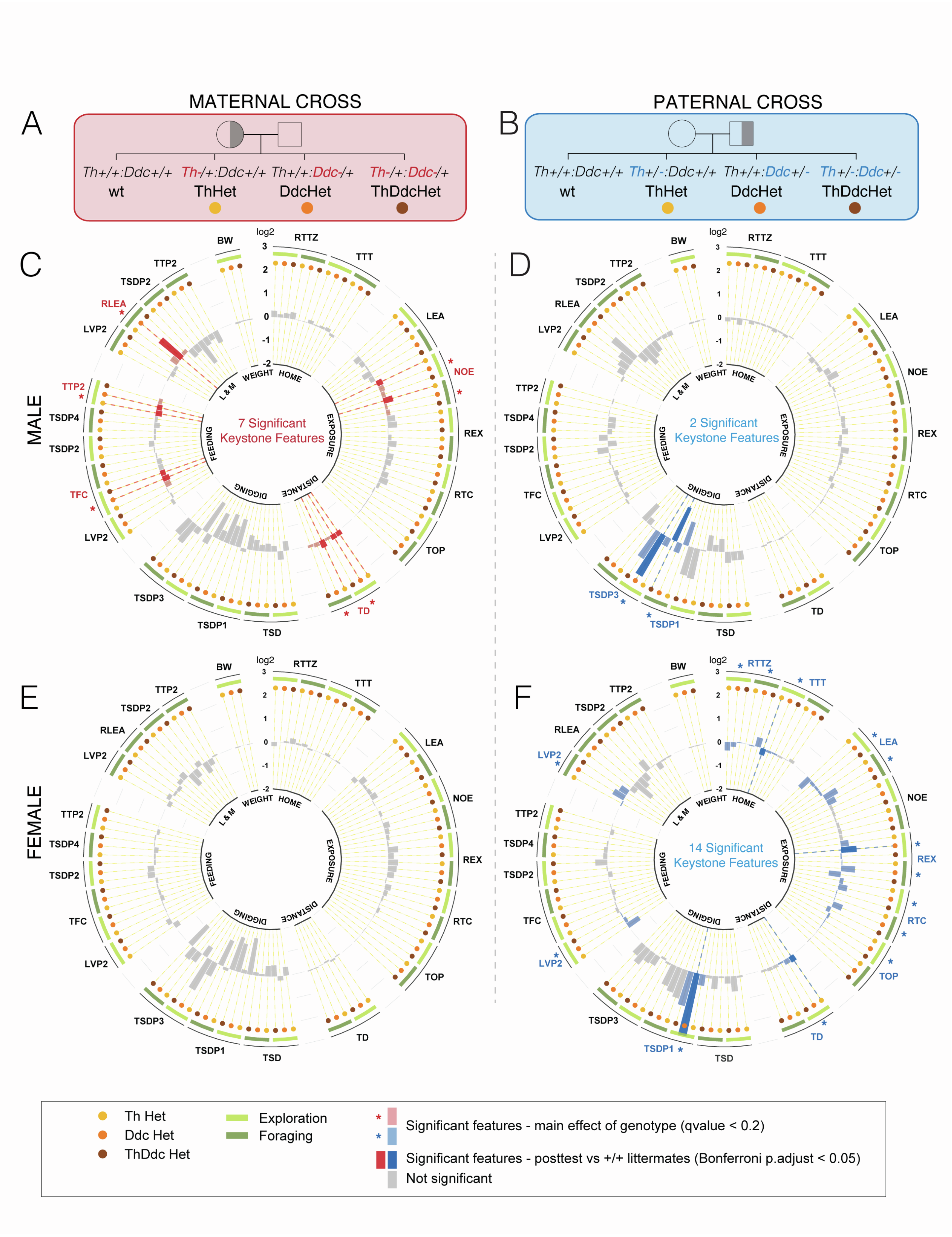

A
MATERNAL CROSS
Th+/+:Ddc+/+ wt
Th-/+:Ddc+/+ ThHet
Th+/+:Ddc-/+ DdcHet
Th-/+:Ddc-/+ ThDdcHet
B
PATERNAL CROSS
Th+/+:Ddc+/+ wt
Th+/-:Ddc+/+ ThHet
Th+/+:Ddc+/- DdcHet
Th+/-:Ddc+/- ThDdcHet
C
MALE
7 Significant Keystone Features
D
2 Significant Keystone Features
E
FEMALE
F
14 Significant Keystone Features
log2
HOME
EXPOSURE
DISTANCE
DIGGING
FEEDING
L & M
WEIGHT
BW
RTTZ
TTT
LEA
NOE
REX
RTC
TOP
TD
TSD
TSDP1
TSDP3
LVP2
TFC
TSDP2
TSDP4
TTP2
RLEA
Th Het
Ddc Het
ThDdc Het
Exploration
Foraging
Significant features - main effect of genotype (qvalue < 0.2)
Significant features - posttest vs +/+ littermates (Bonferroni p.adjust < 0.05)
Not significant

Figure S1. Summary of the Keystone features significantly affected by loss of the maternal versus paternal *Th* and/or *Ddc* alleles. Related to Figure 1 and see Table S2.

(A and B) Shows the breeding strategies to generate offspring with loss of the maternal (A) versus paternal (B) alleles for *Th*, *Ddc* and *ThDdc*, and a legend to guide the interpretation of C-F.

(C-F) Sunflower plots are shown for the Keystone feature data for males (C and D) and females (E and F) with loss of the maternal (red) or paternal (blue) *Th*, *Ddc* or *ThDdc* alleles (see legend). The results are presented as the mean of mutant animals divided by the mean of the wildtype (wt) littermates on the log2 scale. Thus, bars going outwards from 0 indicate an increase in mutants, and bars going inwards indicate a decrease in mutants relative to wt. The Keystone features are organized according to the foraging outcomes that they measure. Features with a significant main effect of allele genotype (+ versus -) are indicated in light red or blue text and bars and by an asterisk (*$p<0.05$, **$p<0.01$). Gene-specific significant effects relative to wt littermates were found by a Bonferroni corrected post-test (p.adjust < 0.05) and are shown by dark colored red or blue bars with dashed lines to the affected genotype indicator key shown in A and B. Grey bars indicate measures that are not significant. The phase that the feature was measured in is shown by light green (Exploration phase) versus dark green (Foraging phase) bars. The total number of significant Keystone features found is summarized in the center. Table S2 provides a description of each Keystone feature. RTTZ, relative time in tunnel zone; TTT, total time in tunnel; LEA, latency to enter arena; NOE, number of excursions; REX, relative exposure; RTC, relative time in center of arena; TOP, time on arena platform; TD, total distance; TSD, total sand dug; TSDP1, total sand dug from Pot1; TSDP3, total sand dug from Pot3; LVP2, latency to visit Pot2; TFC, total food consumed; TSDP2, total sand dug from Pot2; TSDP4, total sand dug from Pot4; TTP2, total time at Pot2; LVP2, latency to visit Pot2; RLEA, Relative latency to enter the Foraging phase arena.

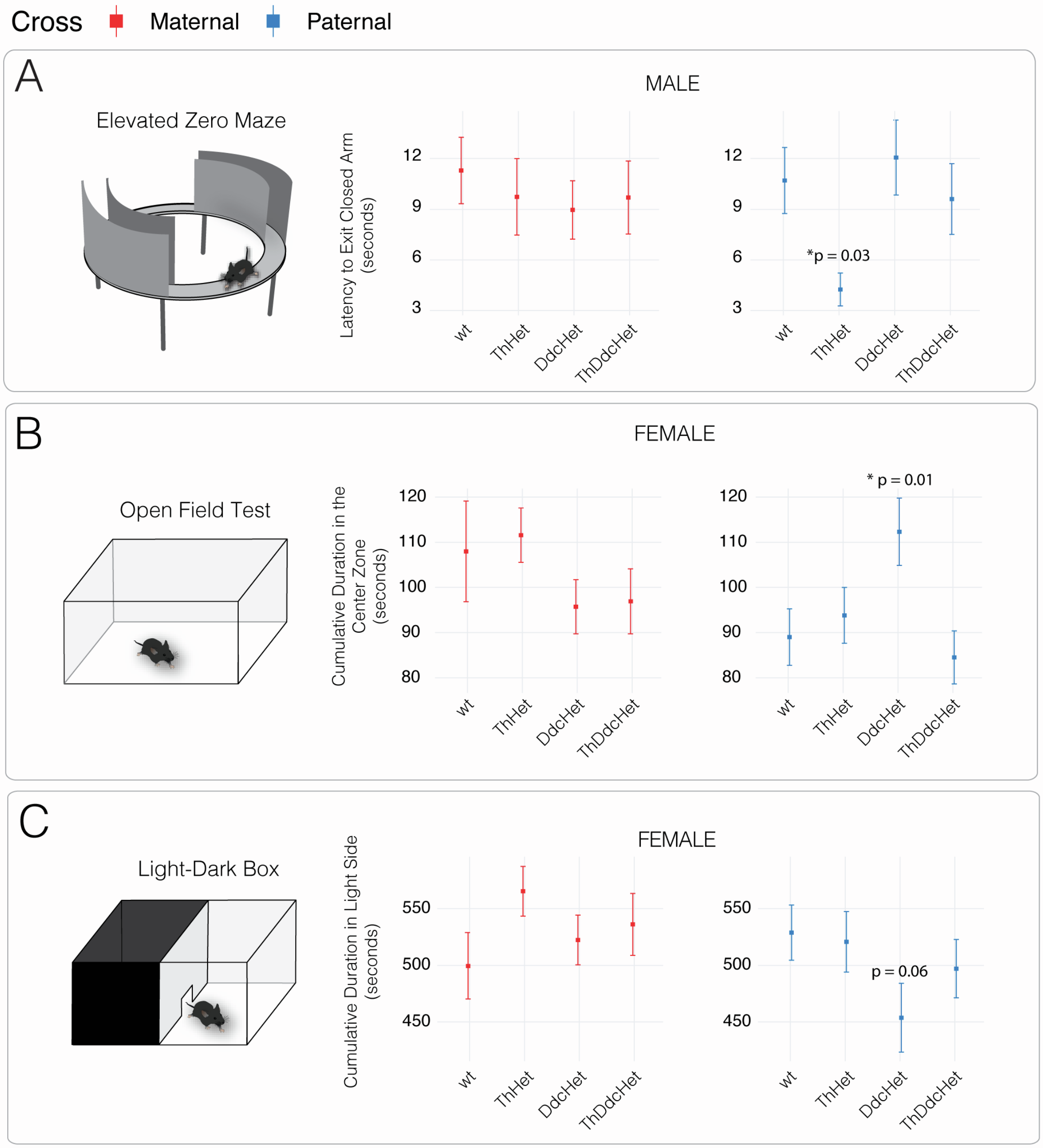
Cross
Maternal
Paternal
A
MALE
Elevated Zero Maze
Latency to Exit Closed Arm (seconds)
12
9
6
3
wt
ThHet
DdcHet
ThDdcHet
*p = 0.03
B
FEMALE
Open Field Test
Cumulative Duration in the Center Zone (seconds)
120
110
100
90
80
* p = 0.01
C
FEMALE
Light-Dark Box
Cumulative Duration in Light Side (seconds)
550
500
450
p = 0.06

Figure S2. Parental effects on standard lab tests of exploratory behaviors and confirmation that *Th* and *Ddc* mutant alleles do not have additive effects in compound *ThDdc* mice. Related to Figure 1.

(A-C) Shows results of measures of exploratory behavior in maternal (red) versus paternal (blue) allele mutants. A, Male *Th* heterozygotes with a mutant paternal allele exhibit a decreased latency to exit the closed arm of the elevated zero maze compared with wildtype (wt) controls, while maternal allele mutants are not significantly affected. The effect in the *Th* mutant is not observed in *ThDdc* compound mutants, showing suppressive effects from *Ddc* similar to our foraging results (n = 20, generalized linear model). B, The duration of time spent in the exposed center zone of the open field test is significantly increased in paternal *Ddc* allele mutant females, but not in maternal allele mutants or other genotypes, and the effect is suppressed in *ThDdc* compound mutants. C, The duration of time spent in the light side of the light-dark phase shows a trend to decrease in paternal *Ddc* allele mutant females and the effect is not increased in *ThDdc* compound mutants. *$p<0.05$.

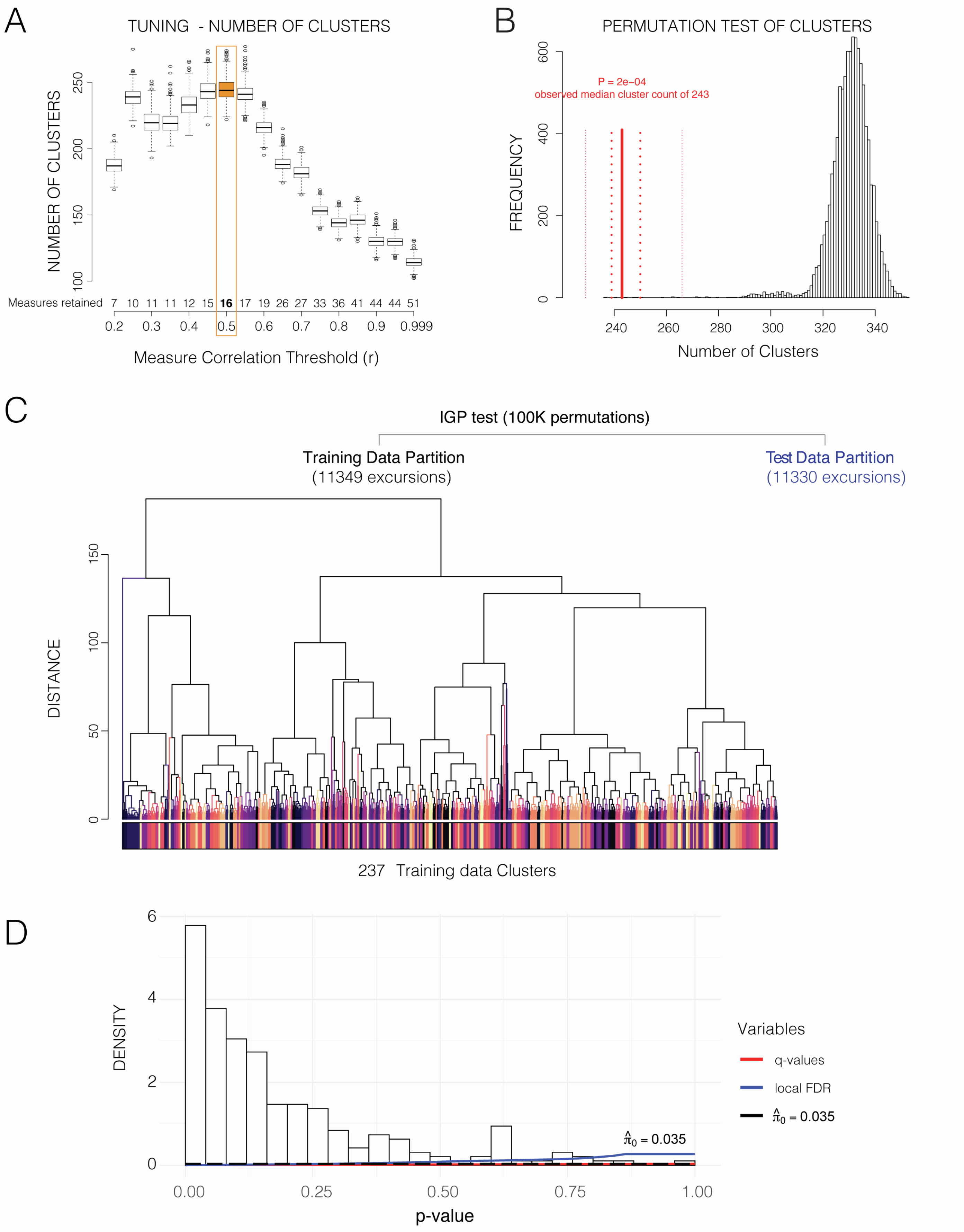
A
TUNING - NUMBER OF CLUSTERS
NUMBER OF CLUSTERS
100
150
200
250
Measures retained 7 10 11 11 12 15 16 17 19 26 27 33 36 41 44 44 51
0.2 0.3 0.4 0.5 0.6 0.7 0.8 0.9 0.999
Measure Correlation Threshold (r)
B
PERMUTATION TEST OF CLUSTERS
FREQUENCY
0
200
400
600
P = 2e−04
observed median cluster count of 243
240 260 280 300 320 340
Number of Clusters
C
IGP test (100K permutations)
Training Data Partition
(11349 excursions)
Test Data Partition
(11330 excursions)
DISTANCE
0
50
100
150
237 Training data Clusters
D
DENSITY
0
2
4
6
0.00 0.25 0.50 0.75 1.00
p-value
Variables
q-values
local FDR
$\hat{\pi}_0$ = 0.035
$\hat{\pi}_0$ = 0.035

Figure S3. DeepFeats identification of foraging behavior modules. Related to Figure 2.

(A) The boxplot shows the number of behavioral sequence clusters found at different correlation thresholds, pruning the 59 measures down to an uncorrelated set that best delineates unique clusters of round-trip foraging excursions from the home. Pruning and clustering is performed 1000 times for each threshold. Retaining 16 measures correlated at $r < 0.5$ maximizes cluster detection and yields stable hierarchical clustering results across different iterations, indicated by a relatively small standard deviation in the numbers of clusters found by dynamic tree cut.

(B) The histogram shows the results of a permutation test to determine whether bona fide clusters are detected from the 16 measures retained at a correlation threshold of $r <0.5$. Dynamic tree cut yields more clusters from randomized data compared to the observed data, consistent with bona fide clustering. The number of clusters detected in the observed data (red line) is significantly less than the number of clusters identified from randomly permuted data (10,000 permutations).

(C) The dendrogram shows the clusters identified in the genotype, phase and sex balanced training partition of the behavioral sequence data by dynamic tree cutting (deep split =4; minimum cluster size = 20). In-Group Proportion (IGP) testing was performed using the centroids from the training data clusters and the excursions in the test data partition.

(D) The histogram shows the q-value analysis results of the IGP test p-values for the training data clusters. At $q<0.1$, 237 training data clusters are significant, which indicates they are reproduced between the training and test data for these clusters of behavioral sequences. We therefore label these as modules.

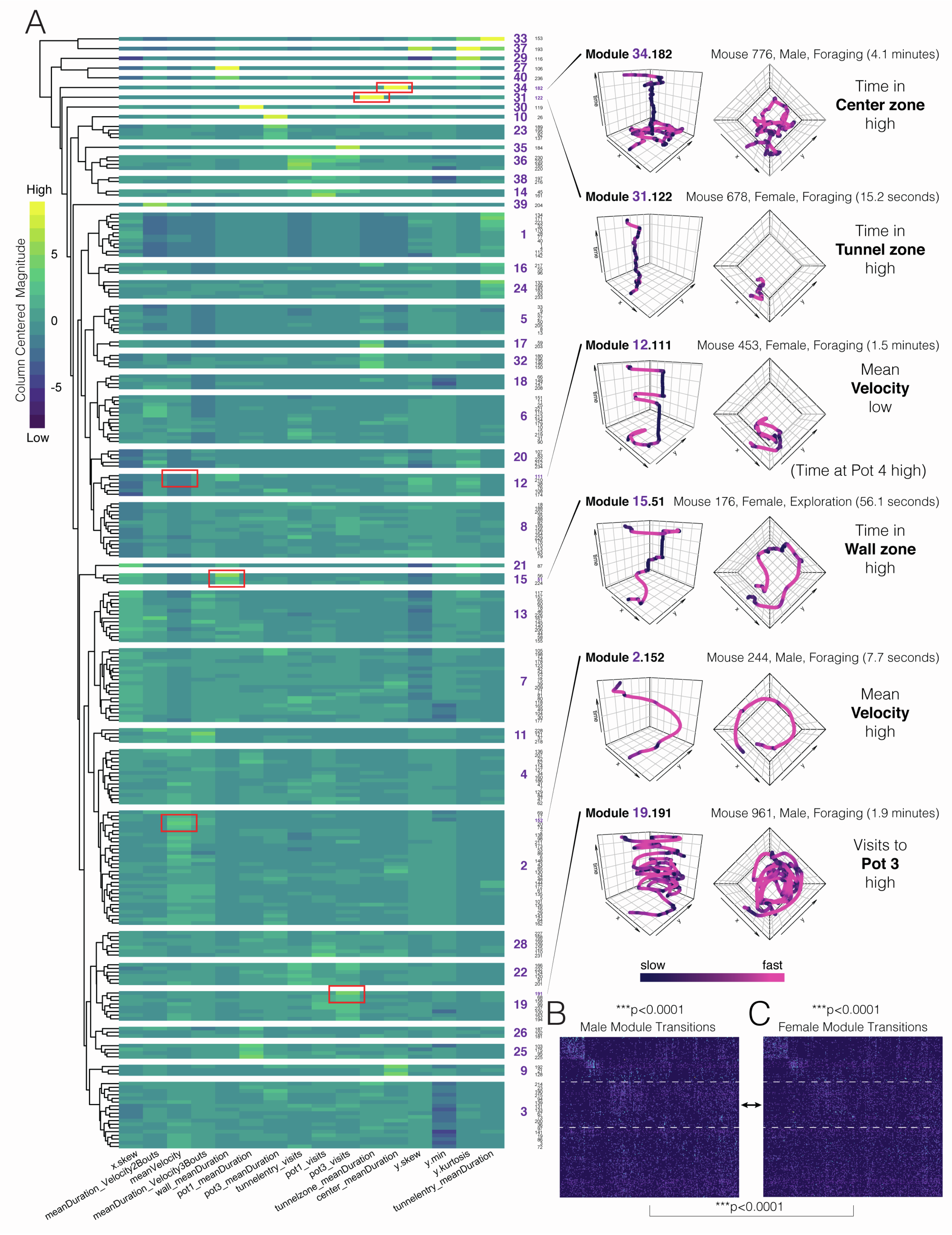

A
High
Low
Column Centered Magnitude
5
0
-5
33
37
29
27
40
34
31
30
10
23
35
36
38
14
39
1
16
24
5
17
32
18
6
20
12
8
21
15
13
7
11
4
2
28
22
19
26
25
9
3
x.skew
meanDuration_Velocity2Bouts
meanVelocity
meanDuration_Velocity3Bouts
wall_meanDuration
pot1_meanDuration
pot3_meanDuration
tunnelentry_visits
pot1_visits
pot3_visits
tunnelzone_meanDuration
center_meanDuration
y.skew
y.min
y.kurtosis
tunnelentry_meanDuration
Module 34.182
Mouse 776, Male, Foraging (4.1 minutes)
Time in
Center zone
high
Module 31.122
Mouse 678, Female, Foraging (15.2 seconds)
Time in
Tunnel zone
high
Module 12.111
Mouse 453, Female, Foraging (1.5 minutes)
Mean
Velocity
low
(Time at Pot 4 high)
Module 15.51
Mouse 176, Female, Exploration (56.1 seconds)
Time in
Wall zone
high
Module 2.152
Mouse 244, Male, Foraging (7.7 seconds)
Mean
Velocity
high
Module 19.191
Mouse 961, Male, Foraging (1.9 minutes)
Visits to
Pot 3
high
slow
fast
B
***p<0.0001
Male Module Transitions
C
***p<0.0001
Female Module Transitions
***p<0.0001

Figure S4. Foraging modules are discrete, reproducible behavioral sequences that construct biologically-valid foraging patterns. Related to Figure 2.

(A) The heatmap shows the centroid data for each of the 237 modules uncovered. Column scaled data for the features (x-axis) that delineate the modules are shown. The modules are assigned identification numbers. Unsupervised clustering of the centroid data revealed groups of modules with similarities, yet consisting of distinct behavioral sequences. Examples of the behavioral sequences for different modules are shown by top and side view tracks. A prominent defining feature in the centroid of each example is shown in text to the right of the tracks. The mouse ID, phase and duration of the behavioral sequence example traces are also indicated. The velocity is plotted by the color in the tracks (see legend).

(B and C) Modules construct biologically valid foraging patterns that differ by sex. The images show the transition probability matrix for module expression in males (B) and females (C). The matrices are calculated using all the module expression data for males or females in the study. The Fisher's Exact Test of independence is statistically significant for both matrices (top, $p<0.0001$). Therefore, state transitions from one module (state 1 module, y-axis) to another (state 2 module, x-axis) are significantly dependent and not random. A permutation test comparing the male versus female transition probability matrices revealed a significant sex difference (bottom, $p<0.0001$). Structure in the matrix data is delineated by white lines. The upper region shows structure in males and females. The middle region shows structure in the male matrix, but not the female matrix (indicated by arrow). Little structure is observed in the lower region.

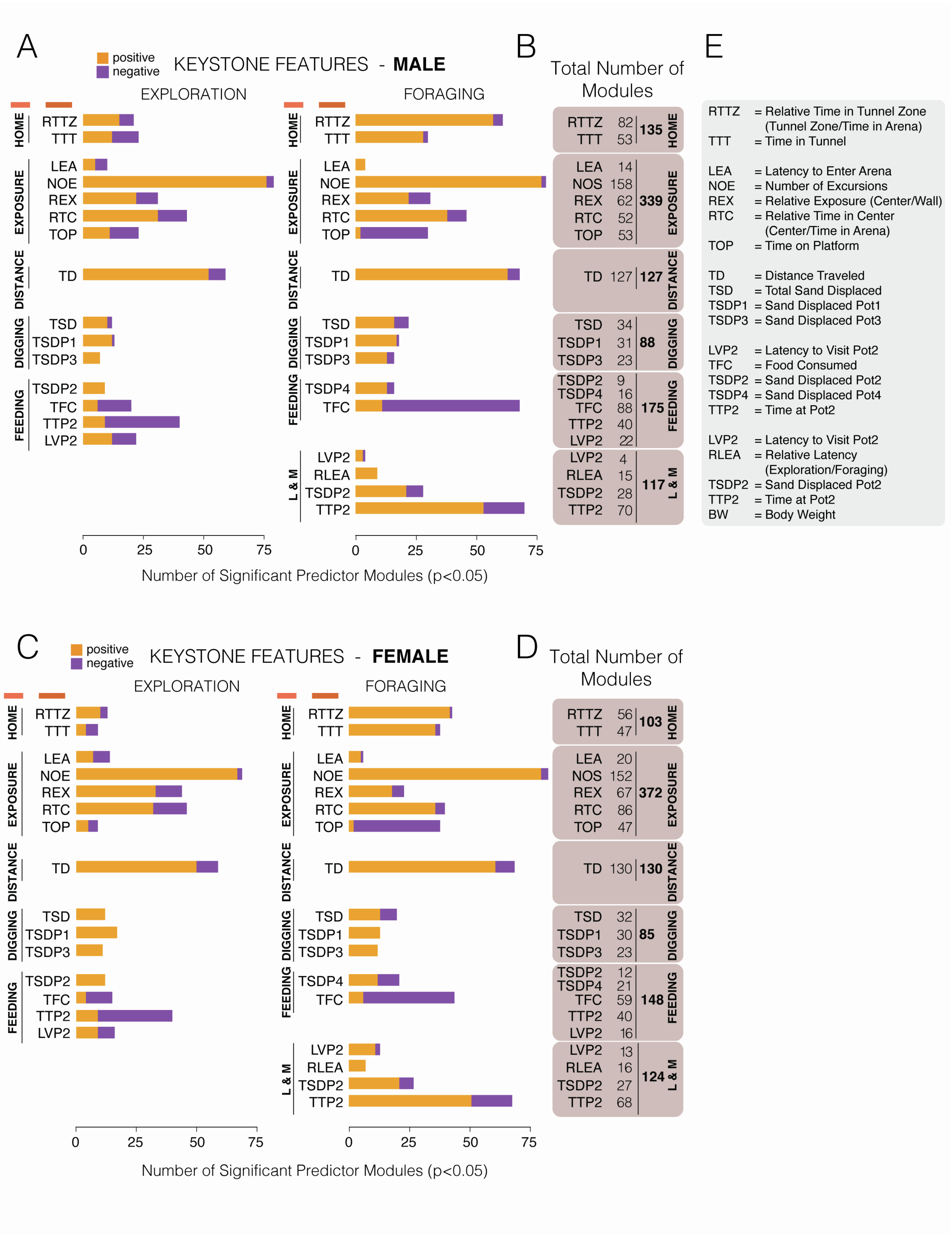
A
positive
negative
KEYSTONE FEATURES - MALE
EXPLORATION
FORAGING
HOME
RTTZ
TTT
EXPOSURE
LEA
NOE
REX
RTC
TOP
DISTANCE
TD
DIGGING
TSD
TSDP1
TSDP3
FEEDING
TSDP2
TFC
TTP2
LVP2
TSDP4
L & M
RLEA
0 25 50 75
Number of Significant Predictor Modules (p<0.05)
B
Total Number of Modules
RTTZ 82
TTT 53
135
LEA 14
NOS 158
REX 62
RTC 52
TOP 53
339
TD 127
127
TSD 34
TSDP1 31
TSDP3 23
88
TSDP2 9
TSDP4 16
TFC 88
TTP2 40
LVP2 22
175
LVP2 4
RLEA 15
TSDP2 28
TTP2 70
117
E
RTTZ = Relative Time in Tunnel Zone (Tunnel Zone/Time in Arena)
TTT = Time in Tunnel
LEA = Latency to Enter Arena
NOE = Number of Excursions
REX = Relative Exposure (Center/Wall)
RTC = Relative Time in Center (Center/Time in Arena)
TOP = Time on Platform
TD = Distance Traveled
TSD = Total Sand Displaced
TSDP1 = Sand Displaced Pot1
TSDP3 = Sand Displaced Pot3
LVP2 = Latency to Visit Pot2
TFC = Food Consumed
TSDP2 = Sand Displaced Pot2
TSDP4 = Sand Displaced Pot4
TTP2 = Time at Pot2
LVP2 = Latency to Visit Pot2
RLEA = Relative Latency (Exploration/Foraging)
TSDP2 = Sand Displaced Pot2
TTP2 = Time at Pot2
BW = Body Weight
C
positive
negative
KEYSTONE FEATURES - FEMALE
EXPLORATION
FORAGING
0 25 50 75
Number of Significant Predictor Modules (p<0.05)
D
Total Number of Modules
RTTZ 56
TTT 47
103
LEA 20
NOS 152
REX 67
RTC 86
TOP 47
372
TD 130
130
TSD 32
TSDP1 30
TSDP3 23
85
TSDP2 12
TSDP4 21
TFC 59
TTP2 40
LVP2 16
148
LVP2 13
RLEA 16
TSDP2 27
TTP2 68
124

Figure S5. Foraging modules are significant predictors of the values of foraging Keystone features. Relates to Figure 3.

(A-D) The barplots show the numbers of modules whose expression significant predicts the values for different Keystone features in males (A and B) or females (C and D) ($p<0.05$, linear model). Response variables in the models include total time in the center of the arena (TTC), total time in the tunnel zone near the home (TTTZ), total food consumed (TFC), total distance traveled (TD), total sand displaced from digging (TSD) and from digging in pot 1 (no food), pot 2 (food, Exploration phase), pot 3 (no food) and pot 4 (food, Foraging phase) (TSDP1-4), and body weight (BW). The total numbers of modules significant predicting each Keystone feature are shown in (B and D). Modules that are positively (orange) or negatively (purple) correlated to Keystone feature values are indicated (Pearson correlation). See text for details. Exp, Exploration phase; For, Foraging phase; r, Pearson correlation.

(E) Shows a legend for the Keystone feature acronyms.

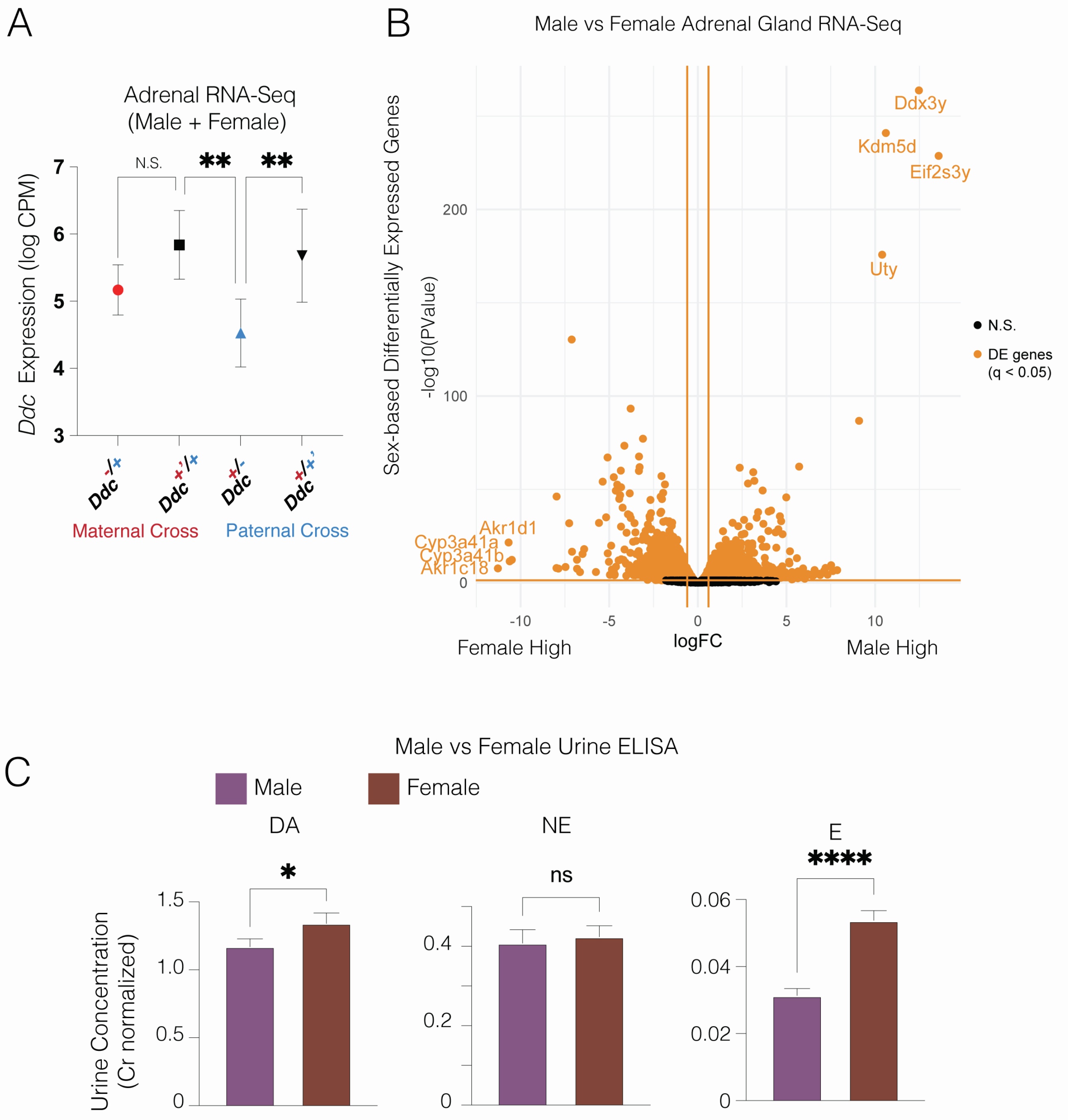
A
Adrenal RNA-Seq
(Male + Female)
Ddc Expression (log CPM)
7
6
5
4
3
N.S.
**
**
Maternal Cross
Paternal Cross
B
Male vs Female Adrenal Gland RNA-Seq
Sex-based Differentially Expressed Genes
-log10(PValue)
200
100
0
Ddx3y
Kdm5d
Eif2s3y
Uty
Akr1d1
Cyp3a41a
Cyp3a41b
Akr1c18
N.S.
DE genes
(q < 0.05)
-10
-5
0
5
10
logFC
Female High
Male High
C
Male vs Female Urine ELISA
Male
Female
DA
NE
E
*
ns
****
Urine Concentration
(Cr normalized)
1.5
1.0
0.5
0
0.4
0.2
0
0.06
0.04
0.02
0

Figure S6. Confirmation of *Ddc* noncanonical imprinting effects and the identification of molecular and endocrine sex differences in the brain-adrenal axis. Related to Figure 6.

(**A**) The plot shows bulk adrenal RNA-Seq expression results for *Ddc* in reciprocal heterozygous *Ddc* mice and +/+ littermates. Significantly decreased expression in paternal allele mutants compared to +/+ wildtype controls are consistent with the imprinting effects observed from our reporter mice (ANOVA, Tukey post-test, n=8). CPM, read counts per millions reads. **p<0.01

(**B**) The volcano plot shows many genes are differentially expressed between males and females in the adrenal gland (orange dots, t-test DESeq2, FDR <5%).

(**C**) The barplots show ELISA detected levels of DA, NE and E in the urine of males versus females normalized to Creatinine (t-test, n=40). Data shows sexually dimorphic DA and E outputs. *p<0.05, ****p<0.00001. Mean$\pm$SEM.

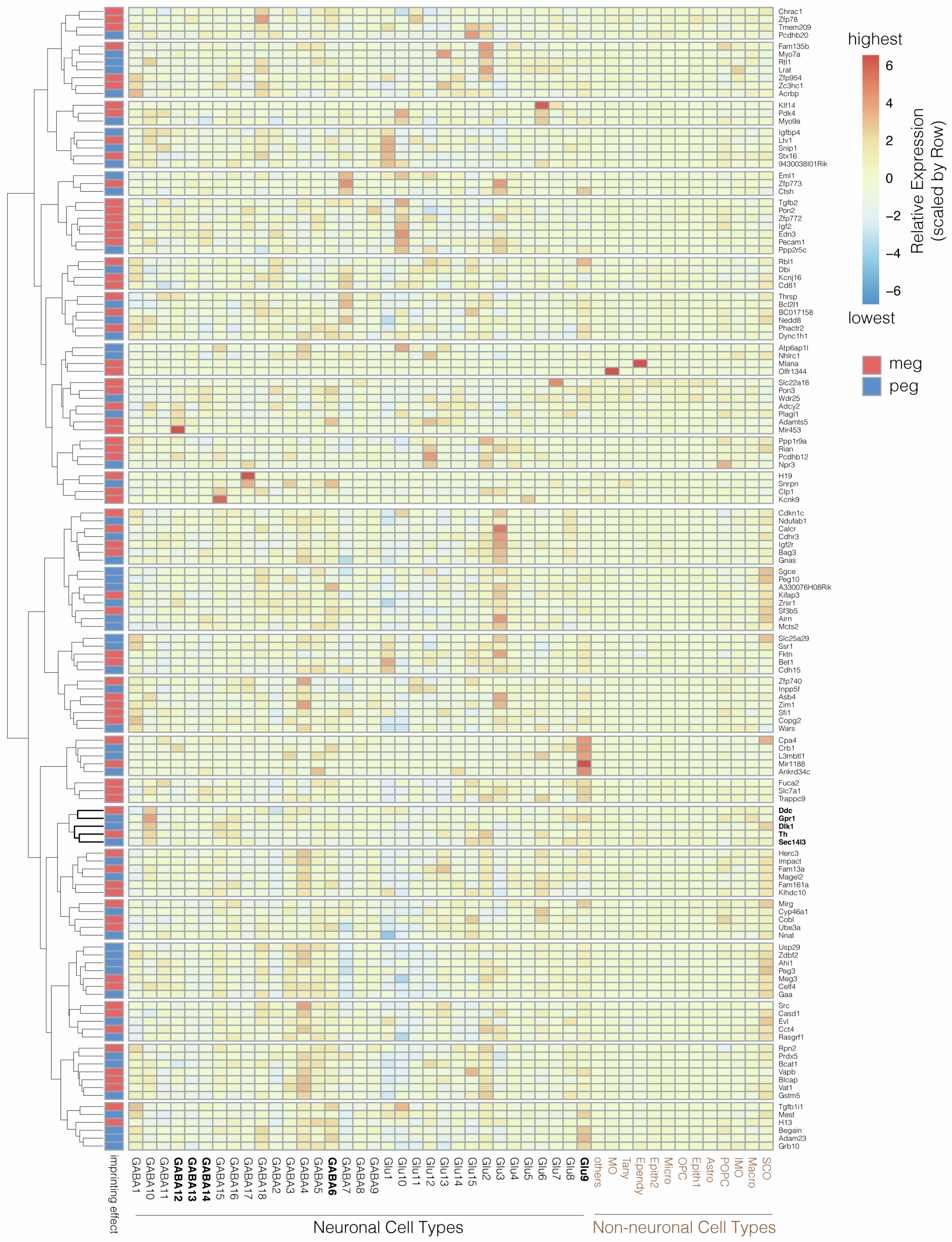
Chrac1
Zfp78
Tmem209
Pcdhb20
Fam135b
Myo7a
Rtl1
Lrat
Zfp954
Zc3hc1
Acrbp
Klf14
Pdk4
Myo9a
Igfbp4
Ltv1
Snip1
Stx16
9430038I01Rik
Eml1
Zfp773
Ctsh
Tgfb2
Pon2
Zfp772
Igf2
Edn3
Pecam1
Ppp2r5c
Rbl1
Dbi
Kcnj16
Cd81
Thrsp
Bcl2l1
BC017158
Nedd8
Phactr2
Dync1h1
Atp6ap1l
Nhlrc1
Mlana
Olfr1344
Slc22a18
Pon3
Wdr25
Adcy2
Plagl1
Adamts5
Mir453
Ppp1r9a
Rian
Pcdhb12
Npr3
H19
Snrpn
Clp1
Kcnk9
Cdkn1c
Ndufab1
Calcr
Cdhr3
Igf2r
Bag3
Gnas
Sgce
Peg10
A330076H08Rik
Kifap3
Zrsr1
Sf3b5
Airn
Mcts2
Slc25a29
Ssr1
Fktn
Bet1
Cdh15
Zfp740
Inpp5f
Asb4
Zim1
Sfi1
Copg2
Wars
Cpa4
Crb1
L3mbtl1
Mir1188
Ankrd34c
Fuca2
Slc7a1
Trappc9
Ddc
Gpr1
Dlk1
Th
Sec14l3
Herc3
Impact
Fam13a
Magel2
Fam161a
Klhdc10
Mirg
Cyp46a1
Cobl
Ube3a
Nnat
Usp29
Zdbf2
Ahi1
Peg3
Meg3
Celf4
Gaa
Src
Casd1
Evl
Cct4
Rasgrf1
Rpn2
Prdx5
Bcat1
Vapb
Blcap
Vat1
Gstm5
Tgfb1i1
Mest
H13
Begain
Adam23
Grb10
imprinting effect
GABA1
GABA10
GABA11
GABA12
GABA13
GABA14
GABA15
GABA16
GABA17
GABA18
GABA2
GABA3
GABA4
GABA5
GABA6
GABA7
GABA8
GABA9
Glu1
Glu10
Glu11
Glu12
Glu13
Glu14
Glu15
Glu2
Glu3
Glu4
Glu5
Glu6
Glu7
Glu8
Glu9
others
MO
Tany
Ependy
Epith2
Micro
OPC
Epith1
Astro
POPC
IMO
Macro
SCO
Neuronal Cell Types
Non-neuronal Cell Types
highest
6
4
2
0
−2
−4
−6
lowest
Relative Expression
(scaled by Row)
meg
peg

Figure S7. Mouse hypothalamus scRNA-Seq data reveals sets of co-expressed MEGs and PEGs in hypothalamic cell-types. Related to Figure 7. The heatmap depicts the mean expression level of each imprinted gene computed from normalized scRNA-Seq read counts for cells categorized to the same hypothalamic cell-type from Chen et al. (2017). Unsupervised hierarchical clustering on the data revealed 25 groups of MEGs and PEGs with similar cellular expression patterns. The data reveal all imprinted genes are expressed in both neuronal and non-neuronal hypothalamic cells. The imprinted gene group containing *Ddc* is highlighted in bold on the y-axis (right side). The data is scaled by row to show relative expression levels across cell-types. POPC: proliferating oligodendrocyte progenitor cell; OPC: oligodendrocyte progenitor cell; NFO: newly formed oligodendrocytes; MO: myelinating oligodendrocyte; Astro: astrocyte; Ependy: ependymocyte; Tany: tanycyte; Endo: endothelial cell; Micro: microglia; Macro: macrophage.

SUPPLEMENTAL DATA FIGURE LEGEND

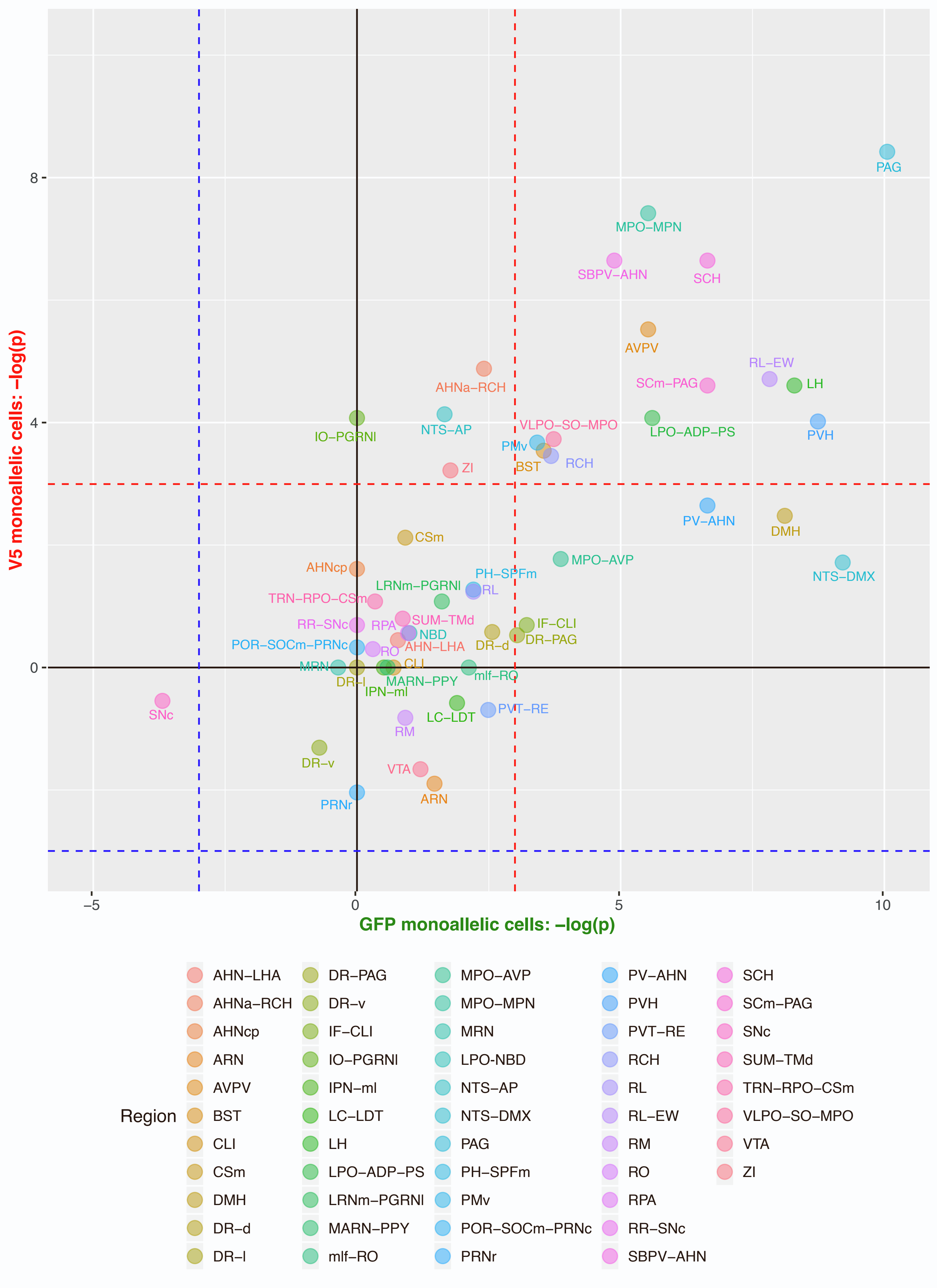

V5 monoallelic cells: –log(p)
GFP monoallelic cells: –log(p)
PAG
MPO–MPN
SBPV–AHN
SCH
AVPV
RL–EW
LH
AHNa–RCH
SCm–PAG
IO–PGRNl
NTS–AP
VLPO–SO–MPO
LPO–ADP–PS
PVH
PMv
ZI
BST
RCH
PV–AHN
DMH
CSm
MPO–AVP
NTS–DMX
AHNcp
PH–SPFm
LRNm–PGRNl
RL
TRN–RPO–CSm
SUM–TMd
RR–SNc
RPA
IF–CLI
NBD
DR–PAG
POR–SOCm–PRNc
AHN–LHA
DR–d
RO
MRN
CLI
DR–l
MARN–PPY
mlf–RO
IPN–ml
SNc
LC–LDT
PVT–RE
RM
DR–v
VTA
ARN
PRNr
Region
AHN–LHA
AHNa–RCH
AHNcp
ARN
AVPV
BST
CLI
CSm
DMH
DR–d
DR–l
DR–PAG
DR–v
IF–CLI
IO–PGRNl
IPN–ml
LC–LDT
LH
LPO–ADP–PS
LRNm–PGRNl
MARN–PPY
mlf–RO
MPO–AVP
MPO–MPN
MRN
LPO-NBD
NTS–AP
NTS–DMX
PAG
PH–SPFm
PMv
POR–SOCm–PRNc
PRNr
PV–AHN
PVH
PVT–RE
RCH
RL
RL–EW
RM
RO
RPA
RR–SNc
SBPV–AHN
SCH
SCm–PAG
SNc
SUM–TMd
TRN–RPO–CSm
VLPO–SO–MPO
VTA
ZI

Data S1. An atlas of adult brain regions containing DDC+ neurons with dominant maternal allele expression. Related to Figure 5.

The scatterplot shows the identity of brain regions containing DDC+ neurons with maternal allele expression confirmed in reciprocal $Ddc^{eGFP/V5}$ and $Ddc^{V5/eGFP}$ crosses and those that do not. Most impacted regions are in the hypothalamus (see legend below). For each brain region, multiple images were captured and blindly scored as having cells with eGFP and/or V5 allele expressing cells. A Fisher's test of a contingency table of the scored images for each region was performed to determine whether a significant number of images had maternal or paternal allele expressing cells or neither (see Methods). Brain regions with significant maternal allele expression are shown (dashed lines show $p<0.05$ threshold). Regions with cells having paternal allele expression in both crosses were not observed. The data show 52 brain regions analyzed by 5 or more optical stacks per region per mouse (n=2, analyzed at 20X magnification). The brain regions are labeled based on Allen Brain Atlas annotations (see Table S2 for brain region definitions).

Tables S5. Primers, targeting constructs and small guide RNAs

Table S5A: Mouse Genotyping Primers

Mice	Primer	Seq. 5'→3'	Band Size (bp)	Genotype
Ddc-6His-GFP	Ddc-5' F<br>Ddc-GFP5' R	CTTGGTTCCATGTCGTCTCCG<br>AACTTGTGGCCGTTTACGTC	303	GFP pos. (5')
	Ddc-GFP3' F<br>Ddc-3' R	GATCACATGGTCCTGCTGGAGT<br>ACATTCTTTCTGCCACTCCTG	298	GFP pos. (3')
	Ddc-5' F<br>Ddc-3' R	*same as above*<br>*same as above*	305<br>1,166	WT<br>homozygous
Ddc-V5-mRuby	Ddc-5' F<br>Ddc-Ruby5' R	*same as above*<br>AACTTGTGGCCGTTTACGTC	303	V5 pos. (5')
	Ddc-Ruby3' F<br>Ddc-3' R	GATCACATGGTCCTGCTGGAGT<br>*same as above*	298	V5 pos. (3')
	Ddc-5' F<br>Ddc-35' F	*same as above*<br>*same as above*	305<br>1,184	WT<br>homozygous
Ddc-Δ7	AADC-S2F<br>AADC-F<br>AADC-R	CCAGCAGCATTGTGGTTTCTAT<br>TTCAGTGTGGGTCTGCCATC<br>CCGAGACCCAAACATCCACA	227<br>349	WT<br>Δ7 mutations

Table S5B: Pyrosequencing AQ Assay Primers

Gene	SNP Position	Primer		
		Forward	Reverse	Sequencing
Herc3	chr6_58826559	Bio/CATGAAGCTGGTAACCCTGTATAA	GGGTTGCAGTCATGTAGTTGTTAA	TGTCTTCCTTCCCCTTA
Ctsh	chr9_89970600	CTATCCCATTCCTCAGGTATAAGC	Bio/TGGCATTCTCATCGAAGGAC	CCATTCCTCAGGTATAAGC
Ube3a	chr7_66541539	GTCCTGGGTCTGGCTATTTACAA	Bio/TCTCCCAAGTCACGAAAGGTTC	GGGTCTGGCTATTTACAA
Igf2r	chr17_12876894	TCTGCTTTCACCGCCTTGG	Bio/GAATGAGACAGAATGGCTGATGG	CCTTGGTGGTGATATGG
Zfp740	chr15_102044608	Bio/GCCAGGGCTTCTGAACATGTA	CCCCAAGGTCTCCCTTAAGAATC	AAGTTATCTCACTTTAGAAA
Eif2c2	chr15_72935534	Bio/ TACACGGATACACACACCTGC	TCATGCTAGAGGACACAGTACCA	GCAGTTACCTGGGAAGT
Ppp1r9a	chr6_4856733	TATAACTCAGACTGGGGAGAGACA	Bio/GCAAGCCAACTATCTCCGAGTA	TGAGGAGGAAGACAGTG
Acrbp	chr6_125003677	AACAGAGCAGTCGGAGACCAG	Bio/CCAGACCCGCCTCTGTCC	GAACATAAGCTAGAAGAAGC

Table S5C: Reporter Construct gBlocks, Primers, and Ultramers

DNA/RNA	type	source	Sequence 5'-3'
Ddc40HA-6His-P2A-eGFP-3nls	gBlock	IDT	CAGTGATCTAGCAAGCAGTGTGCTGAGGGCAGAGAAAGAA***CATCACCATCACCATCAC***ggaagcggagctactaacttcagcctgctgaagcaggctggcgacgtggaggagaaccctggacct**GTGAGCAAGGGCGAGGAGCTGTTCACCGGGGTGGTGCCCATCCTGGTCGAGCTGGACGGCGACGTAAACGGCCACAAGTTCAGCGTGTCCGGCGAGGGCGAGGGCGATGCCACCTACGGCAAGCTGACCCTGAAGTTCATCTGCACCACCGGCAAGCTGCCCGTGCCCTGGCCCACCCTCGTGACCACCCTGACCTACGGCGTGCAGTGCTTCAGCCGCTACCCCGACCACATGAAGCAGCACGACTTCTTCAAGTCCGCCATGCCCGAAGGCTACGTCCAGGAGCGCACCATCTTCTTCAAGGACGACGGCAACTACAAGACCCGCGCCGAGGTGAAGTTCGAGGGCGACACCCTGGTGAACCGCATCGAGCTGAAGGGCATCGACTTCAAGGAGGACGGCAACATCCTGGGGCACAAGCTGGAGTACAACTACAACAGCCACAACGTCTATATCATGGCCGACAAGCAGAAGAACGGCATCAAGGTGAACTTCAAGATCCGCCACAACATCGAGGACGGCAGCGTGCAGCTCGCCGACCACTACCAGCAGAACACCCCCATCGGCGACGGCCCCGTGCTGCTGCCCGACAACCACTACCTGAGCACCCAGTCCGCCCTGAGCAAAGACCCCAACGAGAAGCGCGATCACATGGTCCTGCTGGAGTTCGTGACCGCCGCCGGGATCACTCTCGGCATGGACGAGCTGTACAAG***ccaaagaaaaagcggaaagtccccaagaagaaacgcaaagttcccaagaaaaagcgcaaagtc* **Tag**ctagcctcgacgacaAGGTgAGtctcgacgcgatctcACATCTGTTTCCTTGTGGAGGCATCAGGATTCCAGCTCAG
Ddc40HA-V5-P2A-mRuby2-3nls	gBlock	IDT	CAGTGATCTAGCAAGCAGTGTGCTGAGGGCAGAGAAAGAA***GGTAAGCCTATCCCTAACCCTCTCCTCGGTCTCGATTCTACG***ggaagcggagctactaacttcagcctgctgaagcaggctggcgacgtggaggagaaccctggacct**GTGTCTAAGGGCGAAGAGCTGATCAAGGAAAATATGCGTATGAAGGTGGTCATGGAAGGTTCGGTCAACGGCCACCAATTCAAATGCACAGGTGAAGGAGAAGGCAATCCGTACATGGGAACTCAAACCATGAGGATCAAAGTCATCGAGGGAGGACCCCTGCCATTTGCCTTTGACATTCTTGCCACGTCGTTCATGTATGGCAGCCGTACTTTTATCAAGTACCCGAAAGGCATTCCTGATTTCTTTAAACAGTCCTTTCCTGAGGGTTTTACTTGGGAAAGAGTTACGAGATACGAAGATGGTGGAGTCGTCACCGTCATGCAGGACACCAGCCTTGAGGATGGCTGTCTCGTTTACCACGTCCAAGTCAGAGGGGTAAACTTTCCCTCCAATGGTCCCGTGATGCAGAAGAAGACCAAGGGTTGGGAGCCTAATACAGAGATGATGTATCCAGCAGATGGTGGTCTGAGGGGATACACTCATATGGCACTGAAAGTTGATGGTGGTGGCCATCTGTCTTGCTCTTTCGTAACAACTTACAGGTCAAAAAAGACCGTCGGGAACATCAAGATGCCCGGTATCCATGCCGTTGATCACCGCCTGGAAAGGTTAGAGGAAAGTGACAATGAAATGTTCGTAGTACAACGCGAACACGCAGTTGCCAAGTTCGCCGGGCTTGGTGGTGGGATGGACGAGCTGTACAAG***ccaaagaaaaagcggaaagtccccaagaagaaacgcaaagttcccaagaaaaagcgcaaagtc* **Tag**ctagcctcgacgacaAGGTgAGtctcgacgcgatctcACATCTGTTTCCTTGTGGAGGCATCAGGATTCCAGCTCAG
LHA F1	Primer	IDT	atctgtccaaggccaagagc
LHA R1	Primer	IDT	ttctttctctgccctcagcac
RHA F1	Primer	IDT	acatctgtttccttgtggaggc
RHA R1	Primer	IDT	gaccaaagactgccctggaa
PCRII_Topo_DdcRHAF1	Primer	IDT	AGGCTTATAGAAATAGTTTCCAGGGCAGTCTTTGGTCaagggcgaattctgcagatatcc
PCRIITopo_DdcLHA R1	Primer	IDT	CCTGCTGGATATTGAGAACTGCTCTTGGCCTTGGACAGATaagggcgaattccagcacac
**DDC GFP IVTRT F**	Ultramer primer	IDT	atatcggatcccTAATACGACTCACTATAGGGAGTCTGCCCACGTGCAGCTGGCCTGGGAACACATCAGTGATCTAGCAAGCAGTGTGCTGAGGGCAGAGAAAGAA**CATCACCATCACCATCACGGAAGCGGAGCTACTAACTTCAGC**

**DDC mRuby IVTRT F**	Ultramer primer	IDT	atatcggatcccTAATACGACTCACTATAGGGAGTCTGCCCACGTGCAGCTGGCCTGGGAACACATCAGTGATCTAGCAAGCAGTGTGCTGAGGGCAGAGAAAGAA**GGTAAGCCTATCCCTAACCCTCTCCTCGGTCTCG**
**DDC IVTRT R**	Ultramer primer	IDT	GgaatcctgatgcctccacaaggaaacagatgtccctggtgcagctagCttacCTGAACGTCGAGACGAATCAGACTTTGCGCTTTTTCTTGGGAACTTTGCGTTTCTTC
**Ddc RTR**	Primer	IDT	Ggaatcctgatgcctccacaaggaaacagatg
**Ddc crRNA**	Custom RNA	IDT	AAUGAAAGCAGAGCUGCUUC
**trRNA**	RNA	IDT	CAT# 1072534
